# Development and in vitro evaluation of liposomes and immunoliposomes containing 5-fluorouracil and R-phycoerythrin as a potential phototheranostic system for colorectal cancer

**DOI:** 10.3762/bjnano.17.7

**Published:** 2026-01-09

**Authors:** Raissa Rodrigues Camelo, Vivianne Cortez Sombra Vandesmet, Octavio Vital Baccallini, José de Brito Vieira Neto, Thais da Silva Moreira, Luzia Kalyne Almeida Moreira Leal, Claudia Pessoa, Daniel Giuliano Cerri, Maria Vitória Lopes Badra Bentley, Josimar O Eloy, Ivanildo José da Silva Júnior, Raquel Petrilli

**Affiliations:** 1 Federal University of Ceará, Faculty of Pharmacy, Dentistry and Nursing, Department of Pharmacy, Fortaleza, Ceará, Brazil; 2 Federal University of Ceará, College of Medicine, Department of Physiology and Pharmacology, Fortaleza, Ceará, Brazil; 3 School of Pharmaceutical Sciences of Ribeirao Preto, University of São Paulo, Ribeirão Preto, São Paulo, Brazil; 4 Federal University of Ceará, Center of Technology, Department of Chemical Engineering, Fortaleza, Ceará, Brazil

**Keywords:** colorectal cancer, immunoliposomes, photodynamic therapy, R-phycoerythrin, 5-fluorouracil

## Abstract

5-Fluorouracil (5-FU) is the first-line drug for the treatment of colorectal cancer (CRC), which is considered the third most prevalent type of cancer in the world. R-phycoerythrin (R-PE) is a phycobiliprotein isolated from red algae such as *Solieria filiformis*, with fluorescent properties, photodynamic activity and potential for cancer treatment. However, 5-FU toxicity promotes several side effects and R-PE low stability hampers its clinical use. Thus, the present work aimed to develop co-encapsulated liposomes system for co-delivery of 5-FU and R-PE as theranostic nanosystems for CRC, as well as immunoliposomes targeted with the anti-EGFR monoclonal antibody, cetuximab, as a strategy for targeted delivery to EGFR-positive CRC. To the best of our knowledge, this is the first study to report the development and in vitro evaluation of liposomes and immunoliposomes co-encapsulating 5-FU and R-PE. Thus, liposomes containing 25 mg or 50 mg of soybean phosphatidylcholine (SPC), diesterolphosphatidylcholine (DSPC), dipalmitoylphosphatidylcholine (DPPC), hydrogenated soybean phosphatidylcholine (HSPC) with cholesterol (Chol) and 1,2-distearol-*sn*-glycero-3-phosphoethanolamine-*N*-[amino(polyethylene glycol)-2000] (DSPE-PEG 2000) were prepared and characterized. Among the liposomes, those containing HSPC lipid at 50 mg showed a low polydispersity index (PDI) (0.100 ± 0.022), small size (103.43 ± 1.31 nm), and slightly negative zeta potential (−12.23 ± 0.35 mV). The encapsulation efficiency (EE%) was 94 ± 2.4% for R-PE and 42 ± 2.8% for 5-FU. Regarding the stability study, the liposomes maintained vesicle size, PDI and zeta potential values in a stable range. From the choice of the 50 mg HSPC liposome, the immunoliposomes were developed. The selected immunoliposomes, composed HSPC/DOPE/Chol/DSPE-PEG-Mal in a ratio of 64:10:22.2:3.7, were named HSPC IM 07. This formulation presented low PDI (0.185 ± 0.01), small vesicle size (99.45 ± 1.81 nm), negative zeta potential (−14.8 ± 0.81 mV) and antibody conjugation efficiency of 34.4%. Topographical AFM analysis showed that HSPC-IM-R-PE presented significantly higher surface roughness and viscoelastic contrast, indicating successful antibody anchoring. For cell viability in the HCT-116 CCR cell line, the IC_50_ values for immunoliposomes were higher than those for liposomes. Also, for phototoxicity experiments it was found a reduction in IC_50_ for all groups tested. The internalization of R-PE was verified, highlighting a greater internalization in the immunoliposome within 24 h. Thus, the HSPC 50 formulation containing R-PE and 5-FU, functionalized with cetuximab, is a promising alternative for the development of co-encapsulation delivery systems as a phototheranostic nanocarriers.

## 1 Introduction

Colorectal cancer (CRC) consists in malignant neoplasms related to several histologic types along the colon and rectum. In 2020, worldwide, colorectal cancer is the 3rd most prevalent type of cancer, corresponding to around 10% of the total cases of this disease. Regarding mortality numbers, this kind of cancer reaches the 2nd position, having approximately 900,000 deaths per year [1]. A diversity of factors may contribute, to a greater or lesser extent, to CRC incidence such as genetic mutations and lifestyle. The prognosis, in general, depends on the disease stage and gene affected [2–3]. Among the treatment options for CRC, there are oxaliplatin, irinotecan, mitomycin C and fluoropyrimidines, such as 5-fluSousa et al.orouracil (5-FU), in addition to radiotherapy and anti-EGFR monoclonal antibodies, such as cetuximab [2].

Even though these options are classic treatments for CRC and are well known for improving patient survival, they have numerous side effects, such as immunosuppression, nausea, vomiting, diarrhea, neurological, renal, and cardiac damage [4]. In addition, classic chemotherapy faces another challenge, which is multiple drug resistance (MDR), considering that approximately half of metastatic CRC cases are resistant to 5-FU treatments, contributing to decrease the survival rate of patients [5]. In this context, nanotechnology has emerged as a promising therapeutic strategy for cancer treatment. Nanostructured drug delivery systems enable preferential drug accumulation in tumor tissue through the enhanced permeability and retention (EPR) effect, thereby reducing cytotoxic exposure to healthy tissues and minimizing side effects [6]. Liposomes have promising characteristics due to their biocompatibility and their ability to carry both hydrophilic and lipophilic substances. Furthermore, liposomes can also act as a protein delivery system, reducing enzymatic degradation of proteins and enhancing their stability and their permeability through cell membranes [7].

Immunoliposomes provide many advantages by surface functionalization with targeting biomolecules. These systems are formed by monoclonal antibodies linked to the lipid bilayer, allowing the nanoparticle to recognize and specifically bind to receptors overexpressed in the membrane of tumor cells. This active functionalization expands the therapeutic potential of liposomes by promoting greater intratumor accumulation with higher cellular internalization. In addition, the use of immunoliposomes contributes to reducing systemic toxicity, since their delivery is concentrated in the tumor microenvironment, avoiding exposure of healthy tissues [8].

In colorectal cancer, the epidermal growth factor receptor (EGFR) has been widely explored as a therapeutic target, given its high expression in tumors, especially the most aggressive and metastatic ones [9]. Functionalization with anti-EGFR antibodies, such as cetuximab, allows the construction of immunoliposomes capable of selectively recognizing these tumor cells, enhancing the internalization of the system and promoting greater cytotoxic activity compared to conventional therapies [10]. By combining selective cytotoxicity with the potential to label cancer tissue using an imaging probe, EGFR-targeted immunoliposomes represent an integrated approach for more effective, safer, and personalized theranostic treatment of colorectal cancer [11].

R-phycoerythrin (R-PE) is a phycobiliprotein isolated from red algae *Solieria filiformis*, which is cultivated on the Brazilian coast [12]. Several pre-clinical studies have shown that this protein has potential application in cancer treatment, ranging from in vitro cell programmed death induction in the liver, lung, and gastric cancer cells [12] to in vivo reduction in nodule number and liver weight in rats [13]. In addition, R-PE also has a photosensitizing role that can be explored in photodynamic therapy. Another characteristic of this molecule is its fluorescence activity, which is important for use as a diagnosis tool [14]. Recently, our research group has demonstrated the potential of using the fluorescent properties of R-PE in 4T1 (triple-negative breast murine cancer) and PC3 (human prostate cancer) cells [15].

Furthermore, as previously mentioned, 5-FU is a drug used to treat colorectal cancer, an analogue of the uracil nucleotide, capable of inhibiting the synthesis of DNA and RNA, in addition to inhibiting thymidylate synthase. This drug is the first line of the treatment of colorectal cancer, but there are cellular mechanisms of resistance, such as membrane transporters, which lower the therapeutic response rate. However, when the treatment with 5-FU was evaluated, associated with photodynamic therapy using Foslip^®^ as a liposomal system, it was found that this treatment was effective for cells resistant to 5-FU and for those that are not resistant [16].

In this work, the proposal was to develop nanostructured delivery systems with therapeutic and diagnostic activity by co-encapsulating R-PE and 5-FU in liposomes and immunoliposomes. For this, different lipid compositions were evaluated, and the systems were thoroughly characterized. Finally, in vitro experiments in a CRC cell line were conducted for the evaluation of cytotoxicity, photo toxicity, and uptake. Some studies have sought to demonstrate that dual-drug nanosystems can result in synergistic antitumor effects or reduce adverse effects [17]. This can be considered the first report on the use of liposomes and immunoliposomes containing co-encapsulated 5-FU and R-PE, which may represent a promising strategy for theranostics and allow early tumor treatment.

## 2 Materials and Methods

### 2.1 Materials

The Rhodophyte macroalgae *Solieria filiformis* were collected from cultivation ropes positioned about 200 meters from the shoreline. These activities were overseen by the Association of Algae Producers of Flecheiras and Guajiru (APAFG), situated on the western coast of Ceará State, Brazil (SisGen approval: A41C95F and AA3CF48). R-PE was obtained using a previously reported protocol [15]. 5-Fluorouracil, cholesterol (Chol), Sepharose CL-4B, bovine serum albumin, RPMI medium, trypsin, and 3-[4,5-dimethylthiazol-2-yl]-2,5-diphenyltetrazolium bromide (MTT) were obtained from Sigma-Aldrich (St. Louis, MO). 1,2-Dipalmitoylphosphatidylcholine (DPPC), 1,2-disteasteroyl-*sn*-glycero-3-phosphocholine (DSPC), soja 1,2-disteasteroyl-*sn*-glycero-3-phosphocholine (SPC), hydrogenated soy phosphatidylcholine (HSPC), 1,2-distearol-*sn*-glycero-3-phosphoethanolamine-*N*-[amino(polyethylene glycol)-2000], (DSPE–PEG 2000), and DOPE were obtained from LIPOID (Lipoid GmbH, Germany). DSPE–PEG–MAL was obtained from LaySan Bio, United States. Chloroform (CAS 67-66-3) was obtained from Contemporary Chemical Dynamics (Brazil). Dimethyl sulfoxide (DMSO, CAS 67-68-5), was obtained from NEON (Suzano, Brazil). The cell line HCT-116 (ATCC^®^ CCL-247™) was provided by the National Cancer Institute (USA).

### 2.2 Formulation development

#### 2.2.1 Liposomes

Liposomes were prepared by the lipid film hydration method as previously described [18], based on phospholipid/Chol/DSPE–PEG 2000 (70:20:5) with eight different compositions varying the lipid type (HSPC, DSPC, DPPC, SPC) and amount (25 and 50 mg) followed by sonication. In summary, the lipids were solubilized in 5 mL of chloroform and evaporated for 30 min at 65 °C. In the next step, the lipid film was hydrated with 5 mL of phosphate buffer saline (PBS, pH 7.4) during 60 min at 100 rpm and 37 °C or with PBS, pH 7.4 containing 5-FU and/or R -PE, respectively, at final concentrations of 260 μg/mL and 1 mg/mL. Subsequently, the formulations were subjected to an ultrasonic bath for 15 min. To reduce the size of the vesicles, the sonication method with a probe ultrasound was applied (Qsonica Sonicator – Model: Q500) at a 20% amplitude, for 5 min, under an ice bath [19].

#### 2.2.2 Immunoliposomes

Immunoliposomes were developed from the chosen liposome formulation (HSPC/DOPE/Chol/DSPE–PEG–MAL in a ratio of 64:10:22.2:3.7), with adaptations for better antibody conjugation. Cetuximab (2.0 mg/mL) was thiolated using Traut's reagent, in a molar ratio of 40:1 (Traut/cetuximab), in PBS/EDTA buffer (5 mM, pH 8.0), under incubation at 37 °C for 1 h. The excess Traut's reagent was removed by chromatography on a disposable PD-10 desalting column, collecting 1 mL fractions eluted with PBS/EDTA (5 mM, pH 8.0). The cetuximab concentration was quantified by the BCA assay, according to the specifications of the manufacturer, and the fractions containing thiolated cetuximab (fractions 4–6) were combined. For antibody conjugation, DSPE–PEG–MAL was used as a lipid anchor for cetuximab fixation. Immunoliposomes were prepared by the lipid film evaporation method, as previously described. The fractions containing thiolated cetuximab (fractions 4–6) were then combined with the liposomes and the mixture was incubated overnight for 20 h, at room temperature, stirred at 200 rpm. Unconjugated antibodies were removed by chromatography on a Sepharose CL-4B column eluted with PBS (pH 7.4) [19].

### 2.3 Sample characterization

#### 2.3.1 Particle size, polydispersity and zeta potential

The hydrodynamic vesicle size and polydispersity index (PDI) were performed by dynamic light scattering (DLS), and the zeta potential by electrophoretic light scattering (ELS) techniques using the Zetasizer Nano ZS device (Malvern Instruments). The vesicle size and PDI were evaluated with noninvasive backscatter with an incidence angle of 173°, and the zeta potential was determined by the forward scatter (13°) ELS technique. The samples were diluted (1:200) in Milli-Q water. Given that PBS from the original formulation remains associated with the vesicles and maintains their colloidal stability, no additional saline was required during dilution. The measurements were performed in triplicate at 25 °C using an optical 4 mW HeNe at a wavelength of 633 nm. The results (*n* = 3) were expressed as mean ± standard deviation [20].

#### 2.3.2 Encapsulation efficiency

To determine the encapsulation efficiency (EE%) of 5-FU, an indirect method was employed using ultrafiltration technique and quantification by spectrophotometry. To quantify the free/purified drug, a 1000 μL aliquot of the samples was added to a 50 kDa Amicon^®^, and subsequently centrifuged at 3000 rcf/g for 15 min. Then, a 50 μL aliquot collected from the filtrate was pipetted and the volume was made up to 5 mL with PBS buffer pH 7.4 and subjected to filtration through PVDF with a pore size of 0.45 μm. The samples were analyzed in a spectrophotometer at 265 nm [19]. The amount of total 5-FU corresponds to the theoretical amount of the drug present in the formulation. The results obtained were applied to Equation 1:


[1] $$\text{EE}\%=\frac{\left[\text{purified fraction}\right]}{\left[\text{total fraction}\right]}\times 100.$$

For this, a spectrophotometric method was previously validated [21]. Initially, a stock solution of 1.3 mg/mL of 5-FU in PBS buffer pH 7.4 was prepared. After that, dilutions were carried out obtaining the working solutions used to validate the analytical control: 0.25, 0.5, 1.0, 2.0, 4.0, and 5.0 μg/mL in triplicate. The data obtained was analyzed for precision, linearity, limit of detection (LD), and limit of quantification (LQ) based on ICH Q2(R2) on validation of analytical procedures [22].

For R-PE, an indirect method was used to determine the free/purified fraction. To quantify free/purified drug, a 1000 μL aliquot was removed from the sample and eluted on a CL-4B molecular exclusion gel column. As the sample was eluting, a 1 mL aliquot was collected, and 1 mL of PBS pH 7.4 buffer eluent was added. Finally, a total of twenty fractions were obtained. With the free/purified fraction (eluted fractions), quantification was done by fluorescence (excitation at 495 nm, emission range between 515 and 700 nm) using PBS buffer pH 7.4 as the blank and a 5 nm slit. For this, a spectrophotometric method was previously validated [15]. Initially, a stock solution of 0.05 mg/mL of R-PE in PBS buffer pH 7.4 was prepared. After that, dilutions were carried out obtaining the working solutions used to validate the analytical method: 0.5, 2.0, 4.0, 6.0, 8.0, and 10.0 μg/mL in triplicate.

The method for both molecules was analyzed for precision, accuracy, linearity, LD, and LQ based on ICH Q2(R2) on validation of analytical procedures [22]. Linearity was evaluated using three independent series of calibration curves for 5-FU and R-PE. Linearity was assessed by least-squares linear regression (calibration equation and correlation coefficient). Precision and accuracy were determined by the analysis of replicates of different concentrations. The detection limit and the quantification limit were determined from the calibration curve parameters, standard deviation, and slope of the curve.

The amount of total R-PE corresponds to the theoretical amount of the drug present in the formulation. The results of the concentrations obtained were applied to Equation 1.

The drug loading rate (DL%) of the molecules was determined as a percentage based on the mass of the liposomes obtained after lyophilization, according to Equation 2 [23].


[2] $$\text{DL}\%=\frac{\text{Amount of encapsulated molecule}}{\text{Amount of lyophilized formulation}}.$$

#### In vitro release

**2.3.4.1 In vitro release of 5-FU.** To evaluate the drug release profile, 250 μL of each sample was diluted in 7 mL of phosphate buffer (pH 7.4) and incubated in sealed Erlenmeyer flasks under continuous stirring (150 rpm) at 37 °C (*n* = 5). The assay was performed independently for each release interval (0, 0.5, 2, 4, 6, 8, and 24 h). At predetermined time points, aliquots were withdrawn and filtered through an Amicon^®^ tube (50 kDa), in the centrifugal rotation of 3000*g*. The resulting filtrate was subsequently diluted in phosphate buffer (pH 7.4) and analyzed by UV–vis spectrophotometry at 265 nm [19]. The release profiles of free 5-FU solution, 5-FU-loaded liposomes, and 5-FU-loaded immunoliposomes were comparatively analyzed under the same conditions.

**2.3.4.2 In vitro release of R-PE.** Aliquots of the selected formulations with both molecules were diluted in PBS/azide 0.01% w/v buffer (pH 7.4) to a final R-PE concentration of 125 μg/mL and a final volume of 5 mL, and maintained under agitation at 150 rpm and 37 °C. At 1, 2, 4, 6, 24, 48, and 72 h, samples were ultracentrifuged at 18.000 rpm for 10 min. The supernatant was collected and quantified using a micro BCA assay, following the instructions of the manufacturer. The nanoparticle precipitate was re-dispersed in fresh PBS/azide buffer and agitated until the next time point, as previously described [24].

**2.3.4.3 Release profile of R-PE and 5-FU.** The release profile of R-PE and 5-FU from liposomes and immunoliposomes was analyzed using the Korsmeyer–Peppas (Equation 3) model, commonly employed in drug release kinetics studies, using the DDSolver software [24].

The Korsmeyer–Peppas is a semi-empirical model that encompasses release profiles of drugs through polymeric chains when the governing mechanism is a combination of Fickian and non-Fickian mechanisms:


[3] $$Q=k\cdot t^{n}$$

where *Q* is the amount of dose released at time *t*, *k* is the release rate constant, and *n* is the exponent.

#### 2.3.5 Atomic force microscopy

The topographical characterization of liposomal formulations was performed by using atomic force microscopy (AFM) with a Nanosurf^®^ FlexAFM system. The samples analyzed included liposomes and immunoliposomes containing R-PE. Briefly, to avoid vesicle deformation or disruption, liposomes and immunoliposomes were first stabilized by adding 5% glutaraldehyde for 2 h [25]. After fixation, the formulations were diluted at a ratio of 1:750 in deionized distilled water (ddH_2_O). A 3.0 µL aliquot of the diluted sample was deposited onto a freshly cleaved mica substrate, followed by vacuum drying for 15 min at room temperature. The analyses were conducted in air, using the tapping mode to prevent damage to the sample surface, by using a PPP-NCSTAu probe (Nanosensors^®^, Switzerland), with frequency resonance of 125 kHz and spring constant of 5.0 N/m. The scan rate was 1.5 s per line. The images were captured using the Nanosurf C3000i software and subsequently processed and analyzed with the Gwyddion v 2.66 software, which was used for image leveling, coloring, 3D visualization of the specimens, and roughness analysis [26].

**2.3.5.1 Atomic force microscopy roughness calculation.** Roughness analyses were done by using the Gwyddion software. Each *z*-axis (height) of the image was previously processed to level it, which included shifting the minimum data value to zero, mean plane subtraction, and row alignment using the median of the differences function. After these steps, the statistical quantities tool provided in the Gwyddion software calculated single values for *R*_q_ and *R*_a_ (the root mean square roughness and arithmetic mean roughness, respectively) for each image obtained, considering the entire scanned area without any masking. These values and their standard deviation were calculated from [e.g., *n* = 507] HSPC-50-R-PE control liposomes and [e.g., *n* = 788] HSPC-IM-R-PE immunoliposomes, which were obtained from six and eight independent AFM images, respectively.

### 2.4 Fourier transform infrared spectroscopy

Drug–nanoparticle interactions were studied by subjecting the previously lyophilized samples to Fourier-transform infrared (FTIR) spectroscopy in an FTIR spectrophotometer (IRTracer-100, Shimazdzu, Japan), with a horizontal attenuated total reflectance accessory. The scan was performed in the range of 500 to 4000 cm^−1^ [27].

### 2.5 Stability study

After physicochemical characterization, the selected formulations were 30 days. The samples were kept at 4 °C in plastic containers wrapped in aluminum protected from light, and characterized for vesicle size, PDI, and zeta potential.

#### 2.5.1 Stability of nanoparticles in serum

The stability of the nanoparticles in bovine serum was assessed following an adapted method [28]. Liposomes and immunoliposomes (with and without 5-FU and R-PE) were diluted in 10% serum and incubated at 4 °C for 12 h, 24 h, and 48 h. The samples were vortexed and analyzed for vesicle size, polydispersity index, and zeta potential using a Malvern Nanosizer ZS equipment (Malvern Instruments, UK) at the corresponding incubation times [28].

### 2.6 Phototoxicity and cytotoxicity

The HCT-116 cell line was cultivated in RPMI 1640M medium, supplemented with 10% Fetal Bovine Serum (FBS), and 1% antibiotic/antimycotic solution at 37 °C with 5% CO_2_, in accordance with ATCC recommendations.

The photocytotoxicity and cytotoxicity were evaluated by 3-[4,5-dimethyl-thiazol-2-yl]-2,5-diphenyltetrazolium bromide (MTT) assay, which assesses cellular metabolic activity by quantifying mitochondrial dehydrogenase activity in viable cells, serving as an indicator of cell viability and, consequently, cytotoxicity [29]. For this, the cells were plated onto 96-well plates with 7 × 10^4^ cells per well at 37 °C in 5% CO_2_. After 24 h, the cells were treated with the formulations: liposome (HSPC/Chol/DSPE–PEG2000; 70:20:5; 50 mg of HSPC) and immunoliposome (HSPC/Chol/DOPE/DSPE–PEG–MAL; 64:10:22.2:3.7) containing 5-FU, R-PE, 5-FU/R-PE co-encapsulated and blank formulations. R-PE and/or 5-FU and RPMI medium were used as control solutions. The treatments were diluted in incomplete RPMI medium to obtain curve concentrations according to Table 1.

**Table 1:** Concentration for the MTT method of control and test samples: solution, liposome, and immunoliposome with 5-FU and/or R-PE.

Molecule	Concentration (µM)

5-FU	50.0; 25.0; 12.5; 6.25; 3.15; 1.56
R-PE	0.1; 0.05; 0.025; 0.012; 0.006; 0.003

The microplates were incubated at 37 °C in the presence of 5% CO_2_, for 69 h for cytotoxicity assays, while for phototoxicity assays they remained incubated for 48 h, followed by 2 h of green light irradiation, γ = 525 nm, dose of 14.4 J/cm^2^ and incubated again until completing the 69 h period. After incubation for 69 h, 20 µL of the MTT solution (0.5 mg/mL) was added, followed by incubation for 3 h (for a total 72 h of incubation) at 37 °C. After this period, the medium containing MTT was discarded from the wells and DMSO was added to dissolve the formazan crystals. Finally, the colored solution was quantified by measuring the absorbance at 562 nm on a plate spectrophotometer (Nivo-Perkinelmer). The cellular viability was calculated as the percentage of viable cells compared to the control group and half maximum inhibitory concentration (IC_50_).

#### 2.6.1 Competitive EGFR-binding of anti-EGFR immunoliposome and EGF in HCT-116 cells using flow cytometry

The experimental procedure was based on previous studies [30] with minor modifications detailed below. HCT-116 colorectal carcinoma cells were seeded at a density of 7 × 10^4^ cells/mL in appropriate culture medium and incubated overnight at 37 °C with 5% CO_2_. The following day, the cells were chilled on ice for 10 min and washed with live cell buffer (phosphate-buffered saline supplemented with 20 mM glucose and 1% bovine serum albumin). Cells were then treated with Alexa Fluor™ 488-labeled EGF (Thermo Fisher Scientific, E13345, 2 μg/mL) alone or following a sequential pre-incubation with either anti-EGFR immunoliposomes HSPC IM 07 (25 μg/mL) or cetuximab (Erbitux, 25 μg/mL) for 5 min. Each treatment was followed by a 20 min incubation at 37 °C. After incubation, the cells were washed twice, detached with trypsin-EDTA, centrifuged, and resuspended in a live cell buffer containing DAPI (3 μM) for viable cell selection. Flow cytometry analyses were conducted on a CytoFLEX (Beckman Coulter) using 405 nm and 488 nm lasers to detect DAPI and Alexa Fluor™ 488 signals, respectively. Competitive binding to EGFR was quantified by measuring the cellular fluorescence intensity of Alexa Fluor™ 488.

### 2.7 Cellular uptake

Cellular internalization was assessed by confocal microscopy. For confocal microscopy, 1 × 10^6^ cells were seeded onto 6-well plates containing sterile cover slides and 1 mL of culture medium per well. After allowing cell adhesion overnight, the culture medium was removed and 1 mL of the liposome and immunoliposome nanoparticle suspension (containing R-PE) diluted to a concentration of 1000 nM in serum-free RPMI medium was added. The treatments were kept during 3, 6, or 24 h. After the internalization periods, the cells on the cover slides were rinsed and fixed using a 2% paraformaldehyde solution. For cell nucleus labeling, a DAPI solution (3 mg/mL) and fluoromount-G were used to preserve fluorescence. The slides were photographed using a Leica TCS SP8 confocal fluorescence microscope, using a 40× magnification objective. The wavelengths used were 488 nm for excitation and 575–585 nm for emission of R-PE, and 405 nm with emission between 413–472 nm for DAPI.

The fluorescence intensity quantified from confocal images was performed using the Zeiss Zen 3.12 software integrated with the confocal microscope. For each experimental group, the mean fluorescence intensity and corresponding standard deviation (SD) were determined from the selected regions of interest within the software. The resulting values were exported to GraphPad Prism 8 for statistical analysis.

### 2.8 Statistical analysis

The results were processed using Excel and analyzed in PRISM 8.0 software, and p < 0.05 was considered the minimum value of significance using TwoAway ANOVA test with Tukey's post-test. Competitive EGFR binding assay was analyzed using one-way ANOVA followed by Tukey’s multiple comparisons test.

## 3 Results and Discussion

### 3.1 Liposomes development and characterization

**Figure 1:**
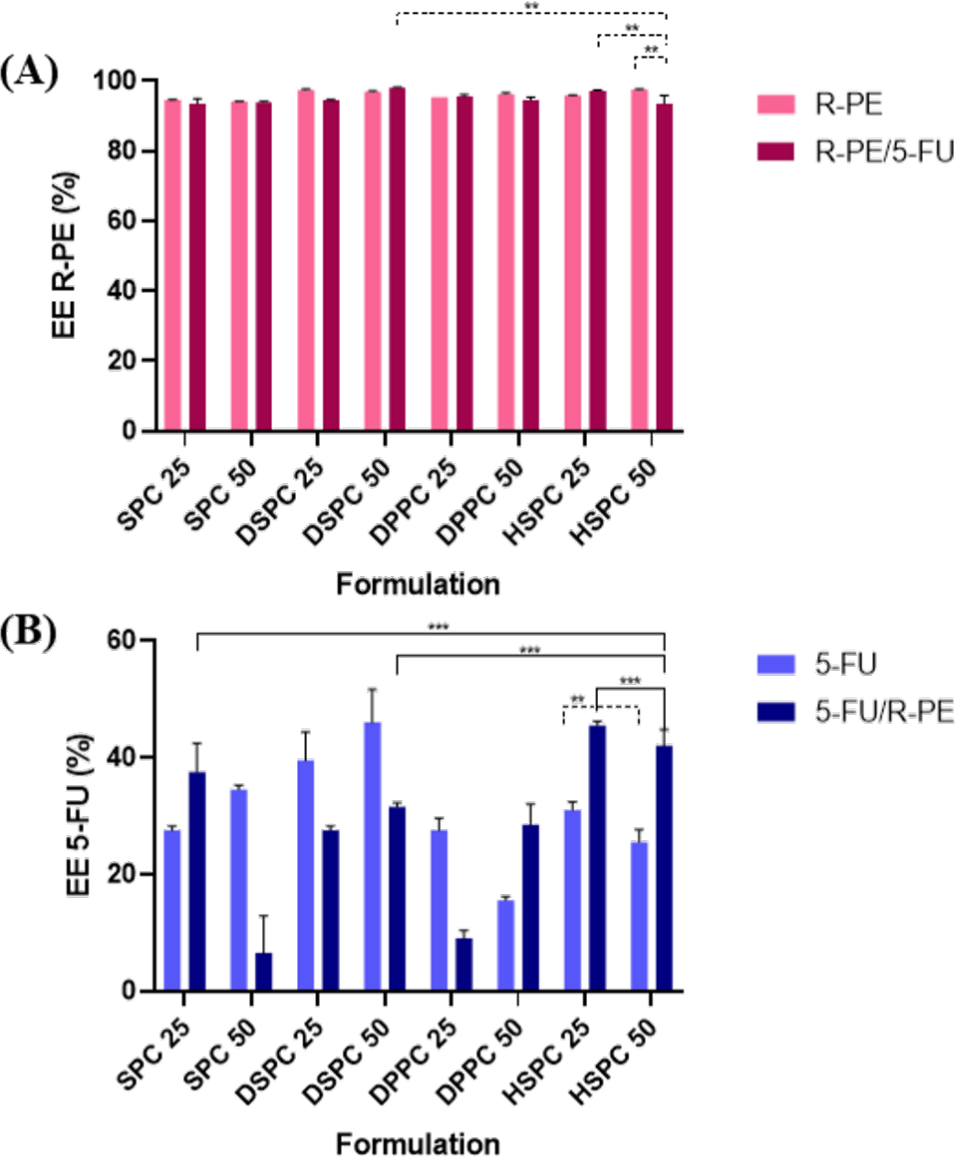
EE% of formulations. (A) Comparison between liposomes containing R-PE alone and those co-encapsulated with 5-FU. (B) Comparison between liposomes containing 5-FU alone and those co-encapsulated with R-PE. (Two-way-ANOVA with Tukey’s test **p < 0.05; ***p > 0.05).

Drug encapsulation in liposomes occurs passively during the formation of vesicles. For water-soluble drugs, such as those used in this study, loading occurs through interactions with the intraliposomal aqueous core. For hydrophilic drugs, encapsulation efficiency tends to be low, so the drug/lipid ratio is usually lower, between 10 and 50% [31]. The EE% results for both 5-FU and R-FE are shown in Figure 1.

The EE% significantly varied depending on the lipid composition and the nature of the encapsulated compound. Formulations containing HSPC exhibited statistically different EE% values for 5-FU and R-PE. However, no significant differences were observed when formulations containing SPC 25 or DSPC 50 were compared to HSPC 50, suggesting that for certain lipid types and concentrations, the EE% may reach a plateau or become less responsive to compositional changes.

A statistically significant difference was observed in the EE% of R-PE between the same HSPC-based formulations. The enhanced encapsulation of R-PE with increasing HSPC content may reflect improved bilayer stability or more favorable partitioning into the aqueous core or thebilayer interface.

In all formulations analyzed, the EE% of R-PE exceeded 90%, indicating a remarkably high level of protein encapsulation. These values are significantly higher than those reported in the literature, where the EE% for the β-subunit of R-PE typically remains around 50% [32]. The high EE% observed in this study may be attributed to the presence of carbohydrates, which are known to reduce pore formation in lipid bilayers and thus enhance vesicle integrity [33]. Such carbohydrates are likely present in the dispersion medium of the liposomes, as the R-PE used was extracted from the red algae *Solieria filiformis*, a species known to produce carrageenan. Additionally, the purity index of the extracted R-PE (0.73) suggests the presence of other biomolecules – such as polysaccharides and residual proteins – which may further contribute to membrane stabilization during liposome formation [15].

For 5-FU, no significant difference was observed between the HSPC 25 and HSPC 50 formulations, which may be explained by small size and high hydrophilicity of the drug – features that hinder its retention in the aqueous core of liposomes. These findings are in line with previous studies: Petrilli et al. (2018) reported EE% values of 45.8 ± 2.0 using DSPC and cholesterol [19], while Crisóstomo et al. (2022) achieved 61.73 ± 0.65 using SPC and cholesterol, which decreased to 50.20 ± 10.20 upon Span 20 addition [34].

Formulations with DSPC showed an improvement in EE%, likely due to its higher phase transition temperature and longer acyl chains, which enhance bilayer rigidity and stability [35]. Literature data support these results, with DSPC/cholesterol (55:45) liposomes showing EE% around 49.9% for 5-FU [21]. The difference in EE% between DSPC 25 and DSPC 50 may be attributed to vesicle size variation, as hydrophilic drug loading is directly related to aqueous volume and vesicle size [36].

Conversely, this trend was not observed in DPPC-based liposomes. DPPC has a lower phase transition temperature (41 °C) than DSPC (55 °C), leading to higher permeability. It has been reported that DPPC liposomes may leak encapsulated compounds even before reaching their transition temperature. Given that film hydration and 5-FU encapsulation were performed at 37 °C, this may explain the reduced EE% observed with DPPC formulations [37–38].

Regarding HSPC, the EE% for 5-FU was 26% for HSPC 50 and 30% for HSPC 25. Although other studies report EE% values of 75–80% for HSPC-based liposomes, those formulations often involve particles up to four times larger than those in the present study [39]. Notably, co-loading of 5-FU and R-PE in HSPC liposomes improved EE% to 46% and 42% for HSPC 25 and HSPC 50, respectively. This suggests a possible interaction between the protein and the drug, enhancing 5-FU retention. Furthermore, the HSPC 50 formulation was the only one to present both high EE% values and a PDI of 0.1 when co-loading both agents, indicating high homogeneity and stability. Thus, HSPC 50 was selected for subsequent immunoliposome preparation, characterization, and efficacy studies against CRC cells.

These findings highlight the importance of optimizing lipid composition not only according to the general hydrophilicity or lipophilicity of the drug, but also considering the molecular size, structure, and specific interactions with lipid components.

**Figure 2:**
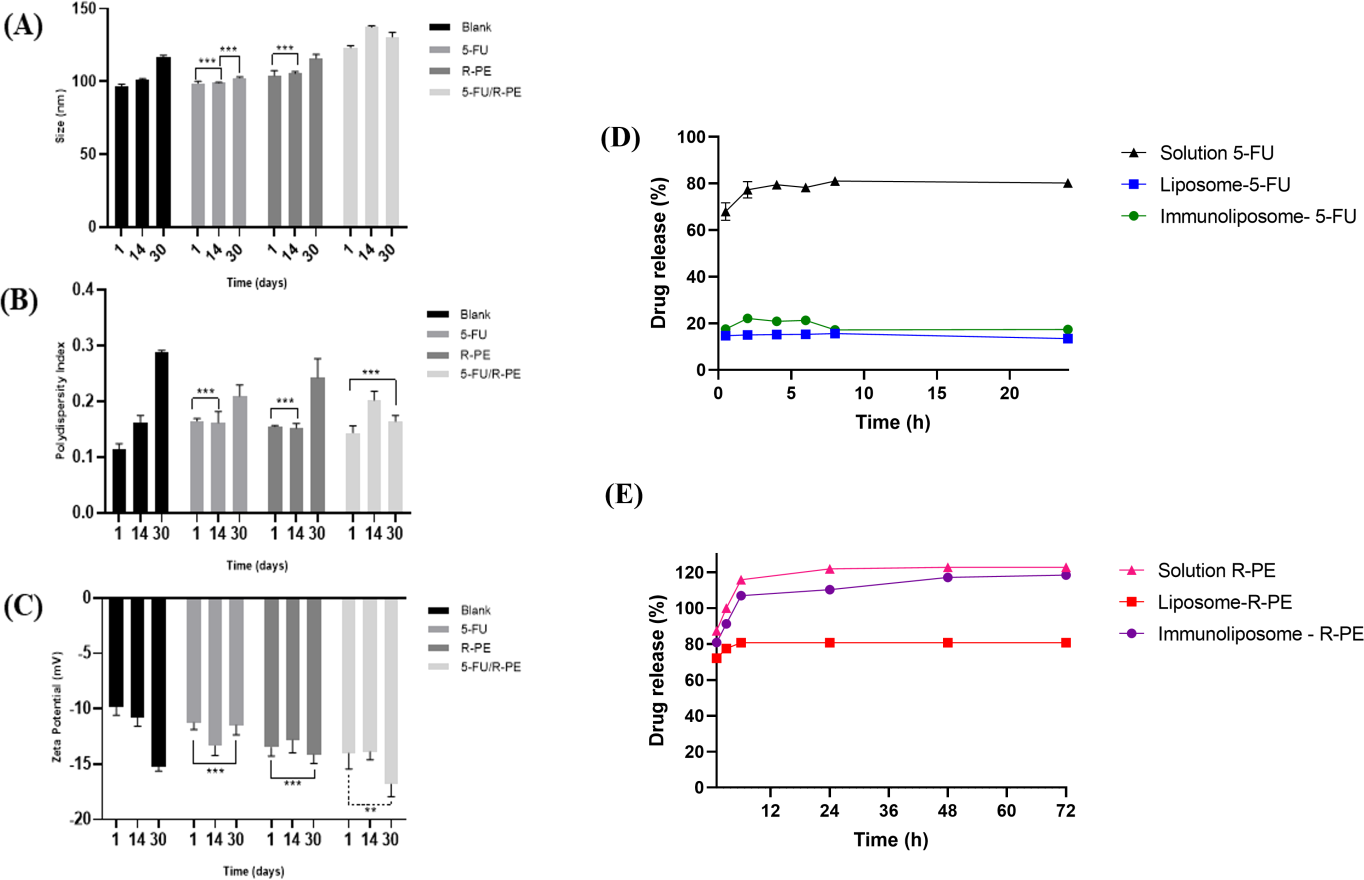
Physicochemical parameters of liposome formulations containing HSPC 50 (with and without 5-FU and/or R-PE) during the stability study at 4 °C for 30 days. (A) Vesicle size, (B) PDI, and (C) zeta potential (Two-way-ANOVA with Tukey’s test **p < 0.05; ***p > 0.05). (D) In vitro release profile of 5-FU from solution, liposome–5-FU, and immunoliposome–5-FU at different time points (0.5–24 h). Data are expressed as mean ± standard deviation (*n* = 3). Statistical analysis was performed by Tukey’s multiple comparisons test: solution vs liposome or immunoliposome, *p* < 0.0001 at all time points; immunoliposome vs liposome, *p* < 0.001 (2 h), *p* < 0.01 (4 h), *p* < 0.001 (6 h), nonsignificant (ns) at other time points. (E) In vitro release profile of R-PE from solution, liposomes, and immunoliposomes in phosphate buffer (pH 7.4) over 72 h (*n* = 3; mean ± SD).

The stability of formulations containing HSPC 50 was evaluated over 30 days (Figure 2A–C). The vesicle size and zeta potential values showed a small increase in relation to storage time, although they are not considered harmful to the stability of the formulation, as they remained in the expected range for liposomes and the PDI remained lower than 0.3. Furthermore, for PDI, no variability was observed when compared with the result of the 1st day of the formulation with 5-FU and R-PE with the last day. Regarding the zeta potential, there was no statistical difference between the days of analysis for samples containing only R-PE and for liposomes with only 5-FU. However, the stability was not shown in the formulations containing 5-FU/R-PE when correlating the zeta potential values from the last day with the previous ones.

Vesicle size is one of the first characteristics to be evaluated in a nanoparticle, as it influences clearance by the mononuclear phagocytic system and affects the drug release rate. Furthermore, smaller particles have greater stability against gravity, due to the Brownian effect [40]. Thus, liposomes around 100 nm are commonly used. Table 2 presents the results obtained herein for different liposomal compositions. The majority of the formulations presented vesicle size smaller than 200 nm, an acceptable value for the intended purpose, with which the EPR effect for therapy is expected tumor [41].

**Table 2:** Physicochemical characterization of liposome formulations, containing Phospholipid/Chol/DSPE–PEG 2000 (70:30:5), where SPC, DSPC, DPPC, and HSPC were used at 25 or 50 mg.

Sample	Blank			5-FU		

	Size (nm)	PDI	ZP (mV)	Size (nm)	PDI	ZP (mV)

SPC 25	82.66 ± 0.20	0.208 ± 0.014	−15.47 ± 1.65	92.06 ± 1.13	0.261 ± 0.003	−10.87 ± 0.75
SPC 50	82.16 ± 0.55	0.190 ± 0.008	−13.47 ± 0.64	81.72 ± 0.71	0.188 ± 0.015	−9.79 ± 1.03
DSPC 25	113.77 ± 1.26	0.134 ± 0.020	−12.20 ± 0.98	124.83 ± 0.50	0.275 ± 0.015	−10.49 ± 0.90
DSPC 50	112.07 ± 1.40	0.153 ± 0.019	−12.77 ± 0.64	133.40 ± 4.90	0.270 ± 0.034	−9.66 ± 1.15
DPPC 25	104.67 ± 0.21	0.157 ± 0.018	−13.80 ± 1.13	94.48 ± 1.77	0.154 ± 0.014	−10.18 ± 0.54
DPPC 50	164.00 ± 2.70	0.309 ± 0.007	−12.07 ± 0.90	100.40 ± 1.52	0.174 ± 0.020	−11.11 ± 1.30
HSPC 25	96.86 ± 0.10	0.160 ± 0.011	−12.03 ± 0.90	116.50 ± 2.31	0.241 ± 0.007	−13.10 ± 1.51
HSPC 50	114.10 ± 1.85	0.159 ± 0.016	−12.27 ± 0.45	113.37 ± 0.81	0.188 ± 0.015	−12.77 ± 0.91

	R-PE			5-FU/R-PE		
	
Sample	Size (nm)	PDI	Size (mV)	Size (nm)	PDI	ZP (mV)

SPC 25	86.22 ± 1.61	0.260 ± 0.002	−22.23 ± 0.45	81.29 ± 0.18	0.202 ± 0.022	−15.03 ± 2.23
SPC 50	84.04 ± 2.09	0.237 ± 0.005	−18.47 ± 0.91	109.63 ± 0.55	0.321 ± 0.030	−16.33 ± 0.81
DSPC 25	141.4 ± 1.11	0.247 ± 0.007	−14.93 ± 1.31	160.57 ± 1.26	0.288 ± 0.010	−13.70 ± 1.51
DSPC 50	148.90 ± 1.06	0.218 ± 0.015	−11.97 ± 0.75	186.80 ± 4.43	0.310 ± 0.040	−13.97 ± 0.61
DPPC 25	120.53 ± 1.20	0.189 ± 0.016	−13.47 ± 1.14	96.87 ± 0.17	0.117 ± 0.014	−18.97 ± 0.61
DPPC 50	176.60 ± 0.56	0.226 ± 0.013	−11.57 ± 0.68	121.63 ± 1.42	0.147 ± 0.018	−11.30 ± 0.56
HSPC 25	132.13 ± 0.91	0.169 ± 0.007	−14.53 ± 0.49	122.30 ± 1.04	0.249 ± 0.007	−14.17 ± 1.19
HSPC 50	125.53 ± 1.82	0.127 ± 0.023	−11.77 ± 0.76	103.43 ± 1.31	0.100 ± 0.022	−12.23 ± 0.35

Based on the results, most formulations showed significant differences. However, no difference was observed in certain comparisons. For SPC 25, the vesicle size values of R-PE and R-PE/5-FU formulations were equivalent to the blank. A similar pattern occurred for HSPC 50 when comparing the blank with the 5-FU-only formulation, and for DPPC 25 both the 5-FU-only and R-PE/5-FU formulations matched the blank. Finally, variations in the proportion of constituents in the blank formulations, such as DSPC and SPC, also did not cause differences in vesicle size.

From the results shown in Table 2, in most cases, a difference in vesicle size values can be observed, depending on the encapsulated molecule. The exception is the formulation that contains the lipid SPC, which generated liposomes around 80 nm. However, SPC 50 formulations with encapsulated R-PE and 5-FU and with encapsulated SPC 25 5-FU were larger, with vesicle size values of 109.63 nm and 92.06 nm, respectively. It was also verified that, in most formulations that contained R-PE, the vesicle size increased. Regarding the vesicle size results of 5-FU with SPC, a study obtained a vesicle size of 97.2 ± 1.25 nm, for liposomes with the same lipid/cholesterol ratio of this work (70:30), similar to those herein (92.1 ± 1.1 nm and 81.7 ± 0.7 nm for SPC 25 and SPC 50, respectively). A slight reduction in vesicle size compared to previous reports can be possibly due to the presence of DSPE–PEG [34].

The incorporation of hydrophilic molecules into liposomal formulations does not necessarily result in an increase in vesicle size [42]. For instance, liposomes composed of HSPC 50 and encapsulating 5-FU did not exhibit a significant change in particle diameter when compared to blank formulations. This behavior may be attributed to the structural instability inherent to liposomes, as their size can vary depending on the lipid composition. Notably, formulations containing HSPC, DPPC, and DSPC exhibited larger vesicle sizes than those composed of SPC. A plausible explanation is that these phospholipids possess a higher bending modulus of the lipid bilayer at room temperature, rendering the membrane less flexible and more rigid compared to SPC, which is known for forming more fluid and deformable bilayers [38,43–44].

The vesicle size distribution graphs obtained by DLS show that the presence of bimodal peaks in the liposome formulations is associated with higher PDI values. For example, the blank formulation of DPPC 50 exhibited a PDI of 0.31, with two distinct populations: one with an average hydrodynamic diameter of 160.9 nm (89.9%) and another of 4401 nm (10.1%). In contrast, the HSPC 50 formulation containing both 5-FU and R-PE showed a monodisperse profile, with a PDI of 0.10 and a mean diameter of 112.8 nm (100%), which is considered satisfactory (PDI ≤ 0.3), indicating a homogeneous particle population [45].

When carrying out a statistical evaluation of the PDI values obtained, in the case of the formulation with 5-FU and R-FE encapsulated in HSPC 50-based liposomes, the lowest PDI value refers to the combination of the two encapsulated molecules when compared with the DPPC formulations, which were not statistically different under the same conditions. Previous reports show that with the DSPC lipid there is greater heterogeneity when compared to DPPC [46].

As expected, all liposomal formulations exhibited a negative zeta potential due to the presence of phosphate groups in the phospholipid headgroups. However, significant differences in zeta potential values were observed between some blank and drug-loaded liposomes, particularly in SPC 25 and DPPC 25 formulations. The presence of R-PE notably influenced the zeta potential, especially in formulations containing SPC 50, DPPC 25, and DSPC 25 and 50. This increase in negative surface charge may be attributed to changes in the distribution of surrounding counterions, possibly caused by the adsorption or ionization of functional groups at the liposomal surface [47]. Additionally, the exposure of the phosphate moiety to the aqueous environment may further contribute to the observed surface charge differences [48].

Among the zeta potential values described in Table 1, only some formulations encapsulated with 5-FU were in the neutrality range, which is −10 to +10 mV. However, some authors consider values greater than −30 to +30 mV to be ideal for the nanoparticle stability to avoid aggregation. The results can be justified by the neutral pH in which these liposomes are dispersed, since in a more acidic medium, it is expected that the amines, present in the lipids, are protonated, decreasing the zeta potential [49].

The DL% rates of the liposome formulations that showed the best performance are described in Table 3. Therefore, the increase in 5-FU encapsulation in liposomes upon adding R-PE was confirmed.

**Table 3:** Drug loading (DL%) of the best performance liposome formulations.

Formulation	DL%

Lipossome 5-FU	0.34 ± 0.03
Lipossome R-PE	5.62 ± 0.01
Liposome 5-FU/R-PE (5-FU)	0.58 ± 0.04
Liposome 5-FU/R-PE (R-PE)	5.05 ± 0.13

### 3.2 Development and characterization of immunoliposomes

Table 4 presents the physicochemical characterization of immunoliposome formulations developed under different preparation conditions, evaluating vesicle size, PDI, zeta potential, and conjugation efficiency. Among the formulations, HSPC IM-07 stands out with the highest conjugation rate.

**Table 4:** Physicochemical characterization of immunoliposomes for vesicle size, polydispersity index, zeta potential, and conjugation efficiency. All formulations contained fixed amounts of cholesterol (7.34 mg) and DSPE–PEG–MAL (10.75 mg).

Formulations	Hspc (mg)	Dope (%)	Temperature (°C)	Agitation (rpm)	Particle size (nm)	Pdi	Zeta potential (mV)	Conjugation efficiency (%)

HSPC IM-01	50	–	37	–	118.83 ± 2.43	0.168 ± 0.018	−22.6 ± 1.04	19.49
HSPC IM-02	50	–	RT	100	127.13 ± 2.81	0.211 ± 0.004	−20.2 ± 1.00	22.46
HSPC IM-03	50	–	37	100	114.9 ± 3.33	0.120 ± 0.006	−19.4 ± 0.16	20.42
HSPC IM-04	50	–	45	–	100.77 ± 2.81	0.133 ± 0.004	−14.9 ± 0.65	12.15
HSPC IM-05	50	–	RT	200	103.86 ± 0.65	0.115 ± 0.005	−16.1 ± 0.57	25.22
HSPC IM-06	50	–	45	200	100.44 ± 2.13	0.126 ± 0.01	−16.1 ± 0.81	19.29
HSPC IM-07	43.25	10	RT	200	99.45 ± 1.81	0.185 ± 0.01	−14.8 ± 0.81	34.4
HSPC IM-08	36.5	20	RT	200	100.44 ± 2.13	0.126 ± 0.01	−16.1 ± 0.81	11.59

The HSPC IM 07 formulation achieved the highest conjugation efficiency, at 34.4%, standing out among the formulations analyzed. This result is likely related to the presence of 10% DOPE in its composition, as well as the preparation conditions – antibody conjugation at room temperature with agitation speed at 200 rpm – which may have contributed to the best conjugation. DOPE promotes rearrangements in the lipid bilayer, increasing its fluidity and consequently facilitating the insertion of ligands onto the surface, in agreement with previous findings [50]. However, increasing the DOPE concentration did not enhance conjugation efficiency. In the IM-D08 formulation, where the DOPE concentration was 20%, the conjugation drastically decreased, indicating that excessive DOPE may compromise the bilayer organization, possibly due to increased instability, thus hindering ligand binding [51].

Moreira et al. (2023) [27] prepared immunoliposomes with DOPE, CHEMS, and DSPE–PEG-PEG to functionalize cetuximab and obtained 14.06% conjugation efficiency with a high percentage of cellular internalization in prostate cancer cells overexpressing EGFR. Souza et al. (2024) [52] also conjugated cetuximab to liposomes based on SPC, cholesterol, and DSPE–PEG–PEG with 40.9% conjugation efficiency.

Regarding vesicle size, HSPC IM 07 presented an average value of 99.45 ± 1.81 nm, within the ideal range, which favors prolonged circulation time and reduced phagocytosis. On the other hand, the polydispersity index was 0.185, slightly higher than that of other formulations, such as IM-D05 (PDI: 0.115) [53]. However, since PDI values up to 0.2 are still considered satisfactory, this parameter is within the expected range [54]. The zeta potential of the selected formulation IM-D07 was −14.8 mV, which is acceptable for ensuring stability, although it does not fall within the ideal range of −20 to −30 mV reported to be more effective for maintaining colloidal stability [55]. The formulations IM-D01 and HSPC IM 02 presented zeta potentials of −22.6 mV and −20.2 mV, respectively, both within the ideal range. However, their conjugation efficiencies were significantly lower – 19.49% and 22.46%, respectively – further supporting the selection of HSPC IM 07 as the optimal formulation.

Another relevant aspect concerns the impact of preparation temperature. A comparison between HSPC IM 05 (room temperature) and HSPC IM 06 (45 °C), both under 200 rpm agitation speed and without DOPE, showed better conjugation efficiency than the formulation prepared at room temperature (25.22% vs 19.29%). This finding is supported by other studies, which demonstrated that elevated temperatures could alter the bilayer structure, negatively affecting ligand orientation and compromising functionalization efficiency [56].

Thus, the HSPC IM 07 formulation exhibits the most favorable characteristics for application in delivery systems, balancing nanoscale size, acceptable PDI, and high conjugation efficiency.

### 3.3 Atomic force microscopy assays

**Figure 3:**
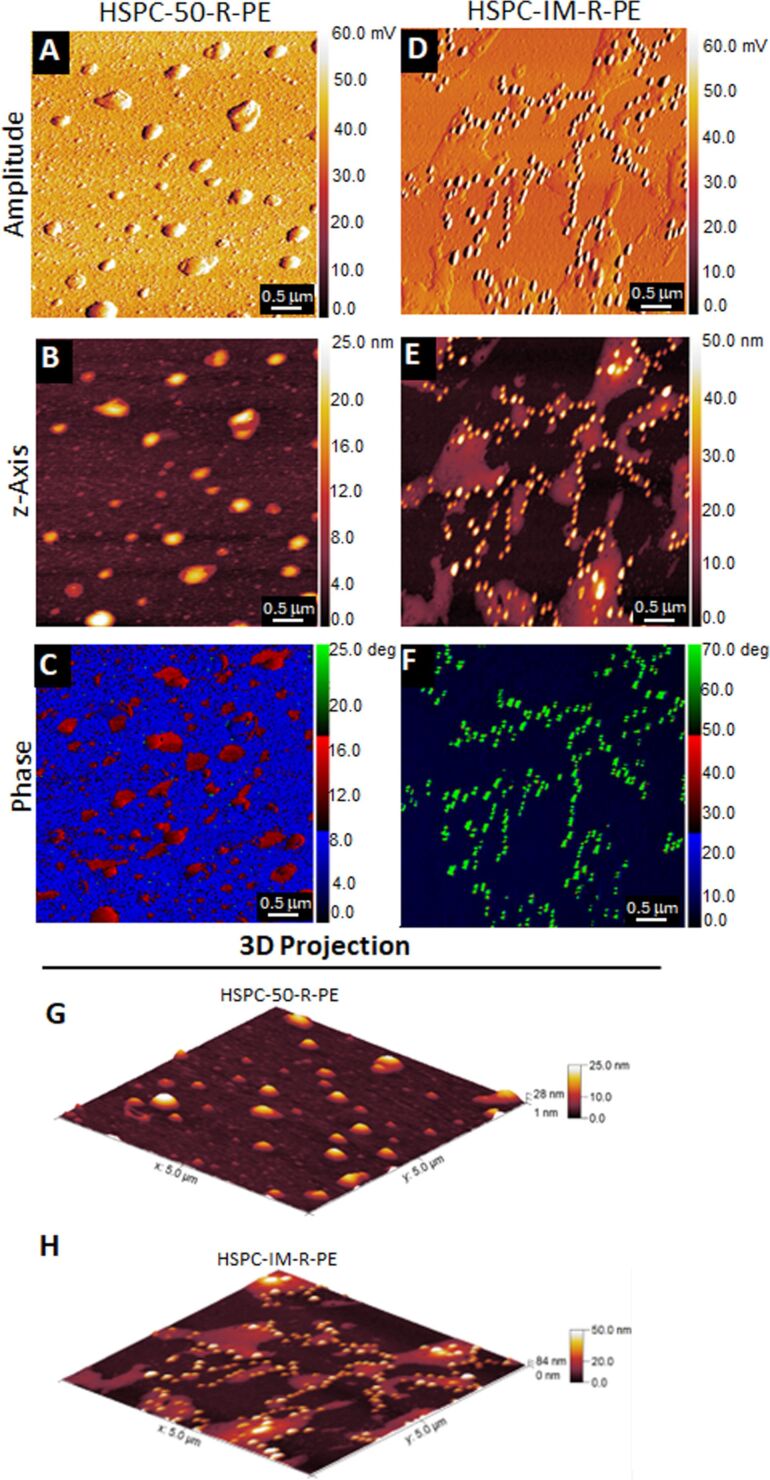
AFM analysis of liposomes (HSPC-50-R-PE) and immunoliposomes (HSPC-IM-R-PE) containing R-PE. Representative AFM images of HSPC-50-R-PE (A, B, C, and G) and HSPC-IM-R-PE (D, E, F, and H). (A, D) Amplitude images showing high-quality imaging without noise artifacts. (B, E) 2D Topographic profile of height (*z*-axis). (C, F) Phase images revealing distinct phase contrast between formulations, see text for further details. (G, H) 3D topographic projections.

**Figure 4:**
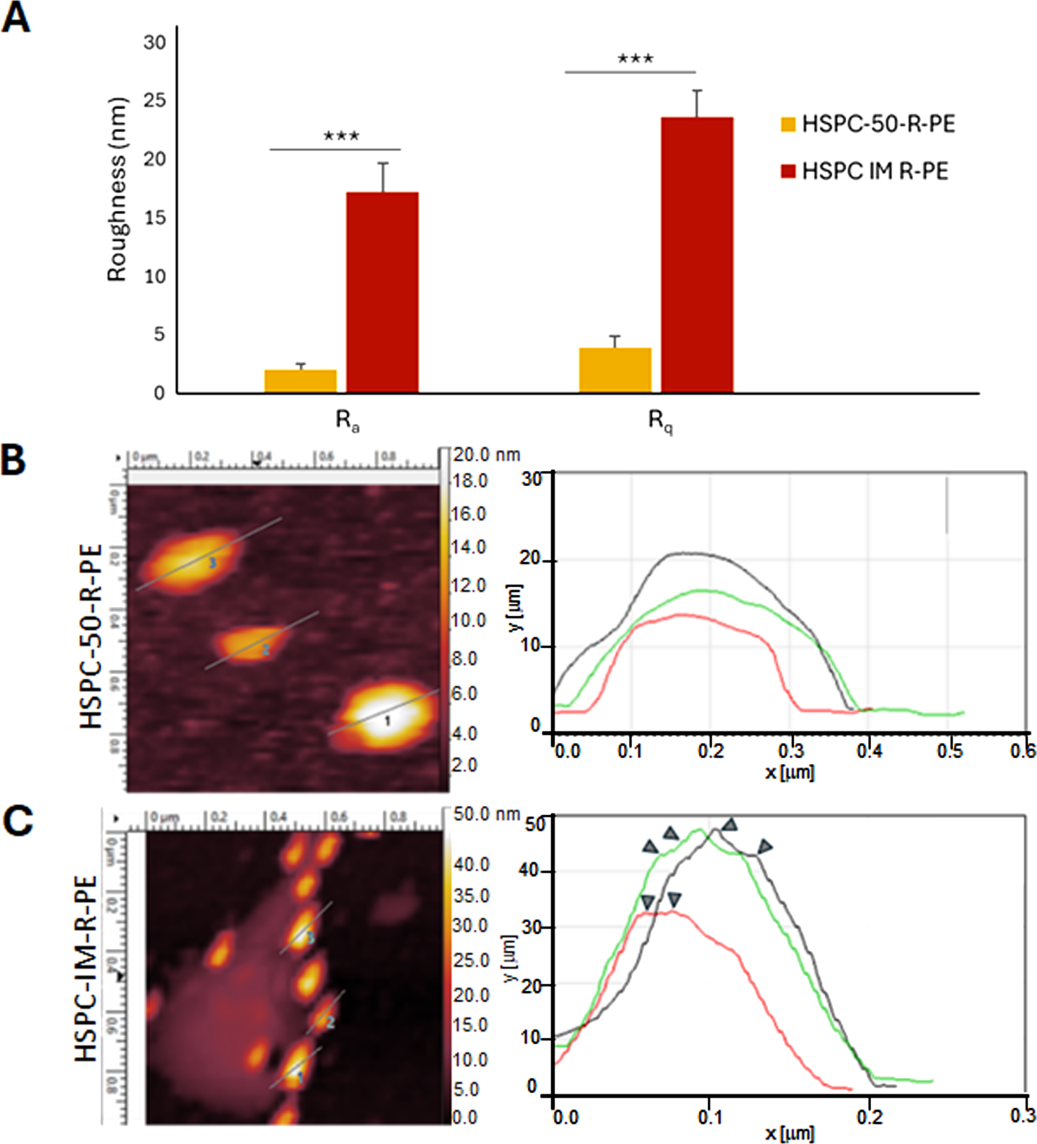
Graphical roughness analysis by AFM of liposomes (HSPC-50-R-PE) and immunoliposomes (HSPC-IM-R-PE) containing R-PE. (A) Quantitative roughness comparison between the two formulations, based on arithmetic roughness (*R*_a_) and root-mean-square roughness (*R*_q_) parameters. (B, C) Profile traces of representative liposomes from each formulation. Arrows in (C) indicate that surface irregularities in HSPC-IM-R-PE are likely resulting from the cetuximab functionalization.

Atomic force microscopy assays were used to characterize the surface topography of nonfunctionalized liposomes (HSPC-50-R-PE) and immunoliposomes (HSPC-IM-R-PE), as shown in Figure 3 and Figure 4. It is worth noting the good quality of the AFM images by analyzing amplitude (Figure 3A and Figure 3D), which presented no noticeable noise artifacts, providing pseudo-3D contrast in both samples. In the case of HSPC-50-R-PE, the *z*-axis and 3D projection images (Figure 3B and Figure 3G, respectively) showed predominantly spherical vesicles, although slightly flattened. This aspect is typical of analyses performed in dried conditions on solid substrates such as mica and is mainly due to the dehydration process and adhesion forces onto the substrate [57–58]. On the other hand, the *z*-axis and 3D projections for HSPC-IM-R-PE (Figure 3E and Figure 3H, respectively) demonstrated a more irregular nanoparticle surface, with visibly rougher and more heterogeneous topography, a result consistent with previous studies [27,59–60].

To complement the topographic data, when comparing phase images of both formulations (Figure 3C and Figure 3F for HSPC-50-R-PE and HSPC-IM-R-PE, respectively), we observe further evidence of consistent structural alterations in the membrane of immunoliposomes, which aligns with the presence of anchored cetuximab on the lipid membrane, promoting changes in the surface organization of the nanoparticle. This is reflected in the relatively low phase angle of approximately 16° for all HSPC-50-R-PE (as indicated by red-colored liposomes) compared to approximately 70° for HSPC-IM-R-PE (as indicated by green-colored immunoliposomes). The lower phase angle indicates a more elastic, stiffer, and homogeneous lipid membrane, whereas the higher phase angle of HSPC-IM-R-PE is consistent with a more dissipative tip–sample interaction, which is characteristic of softer, flexible, and viscoelastic materials [61–62]. The decoration of liposomes with cetuximab may result in a softer outer surface than the underlying lipid bilayer, explaining the observed increase in energy dissipation. These data strongly support that the surface properties of this liposome formulation were indeed altered following antibody functionalization, leading to distinct nanomechanical profiles, further reinforced by the observed changes in surface roughness, as follows.

Herein, we highlighted that AFM roughness measurements were uniquely used to confirm antibody decoration onto the liposome surface, with no correlation to nanoparticle size obtained by DLS, since both assays were performed in different conditions (hydrodynamic vs dried samples for DLS and AFM, respectively). The surface roughness showed a significant difference when both formulations were compared (Figure 4A–4C). Herein, we used the arithmetic roughness (*R*_a_) and the root-mean-square roughness (*R*_q_) as parameters to evaluate surface features [26]. Regarding *R*_a_, as shown in Figure 4A (left pair of bars), the nonfunctionalized liposomes exhibited a notably low value of 2.016 ± 0.5081 nm, indicating a more uniform surface. In contrast, immunoliposomes exhibited a significantly higher *R*_a_ of 17.22 ± 2.487 nm (*p* = 0.0002 vs liposomes). This increase in *R*_a_ is a direct consequence of successful cetuximab conjugation onto the liposomal surface, which introduces irregularities and elevated features, as previously described in proteoliposome membranes [25,59]. This difference is also evident in the profile analysis of selected nanoparticles (Figure 3J and Figure 3K). Irregularities are clearly visible only in HSPC-IM-R-PE (Figure 4C, arrowheads in the right panel), which can be due to cetuximab decoration. In addition to the *R*_a_ results, the *R*_q_ values also support these findings (Figure 4A, right pair of bars). HSPC-50-R-PE maintained a low *R*_q_ of 3.874 ± 0.9706 nm, reinforcing the existence of a smooth topography without extreme height variations. In contrast, HSPC-IM-R-PE reached 23.61 ± 2.225 nm (*p* < 0.0001 vs liposomes), suggesting more distinct surface peaks and valleys, consistent with the functionalization process, which increase roughness and significant topographical variations caused by the conjugated biomolecules. As seen above, the differences between formulations were statistically significant, it is noteworthy that when comparing *R*_a_ and *R*_q_ within each liposome formulation, no statistical significance was observed (HSPC-50-R-PE, *p* = 0.1208 vs *R*_q_; HSPC-IM-R-PE, *p* = 0.0761 vs *R*_q_), indicating sample uniformity within each group.

Taken together, the topographic data, phase angle variations, and quantitative roughness analyses provide strong evidence of liposome functionalization. The transition from a smooth surface in HSPC-50-R-PE to a rougher HSPC-IM-R-PE topography is likely to impact biological interactions such as cellular uptake mechanisms, biodistribution, clearance by the reticuloendothelial system, and even protein corona formation under physiological conditions [63]. These findings highlight the importance of surface characterization by AFM in the development and optimization of nanoparticles.

### 3.4 Fourier-transform infrared spectroscopy assays

**Figure 5:**
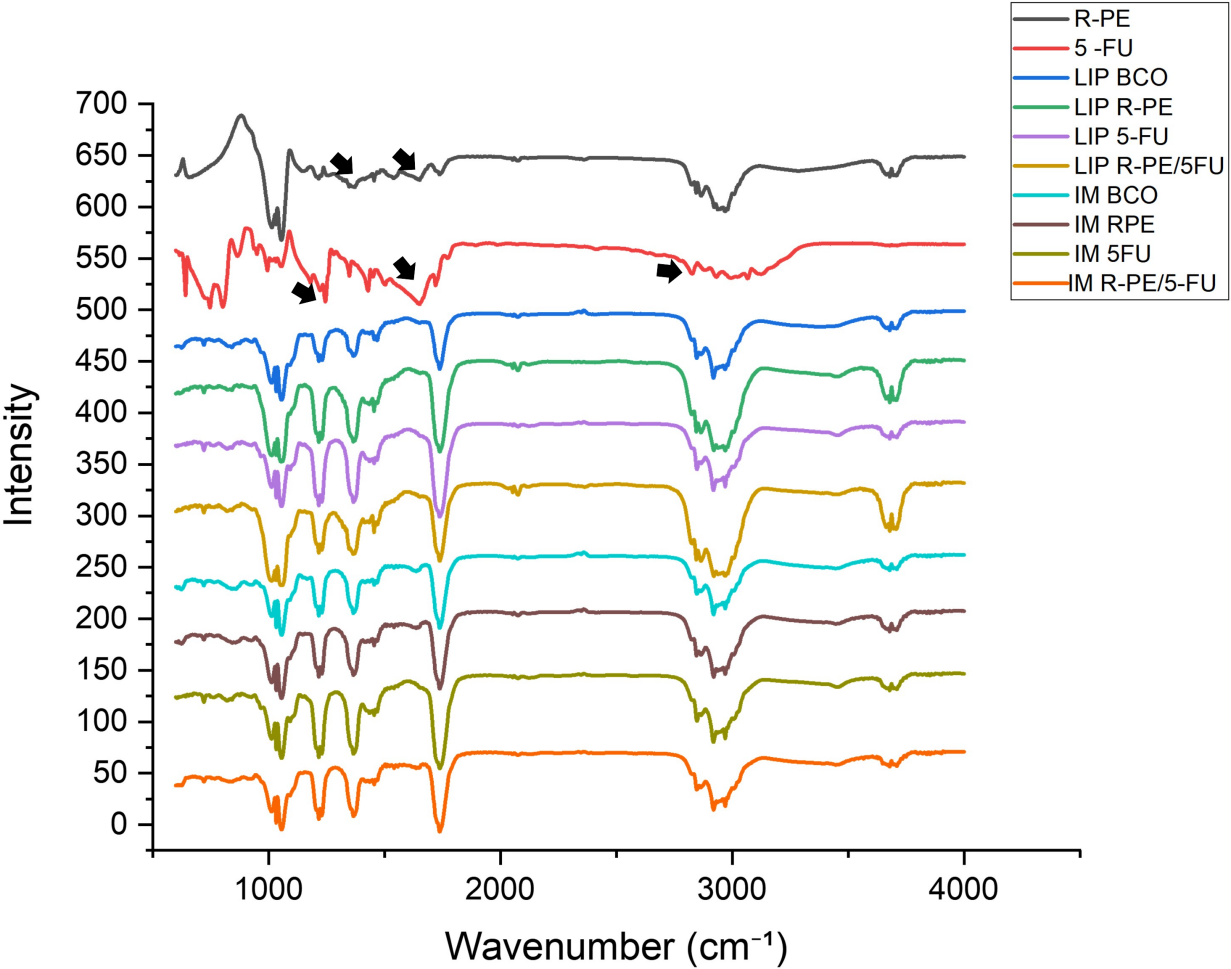
FTIR spectra of R-PE, 5-FU, liposomes, and immunoliposomes. Characteristic bands of R-PE were identified, including amide I (≈1650 cm^−1^), amide II (≈1540 cm^−1^), and the broad N–H/O–H stretching between 3200–3400 cm^−1^. In the spectra of 5-FU, typical vibrations of C=O (≈1670 cm^−1^), N–H (≈3120 cm^−1^), and C–F (≈1240 cm^−1^) were observed.

The FTIR spectra obtained for the formulations showed the characteristic bands of both R-PE and 5-FU, confirming their incorporation into liposomes and immunoliposomes (Figure 5). For R-PE, typical amide vibrations were observed: amide I (C=O) around 1650 cm^−1^ and amide II (N–H) near 1540 cm^−1^, along with a broad N–H/O–H stretching between 3200–3400 cm^−1^, attributed to the polypeptide backbone of the protein. For 5-FU, bands associated with C=O stretching (≈1670 cm^−1^), N–H stretching (≈3120 cm^−1^) [64–66], and the characteristic C–F vibration (≈1240 cm^−1^) were also detected [66].

When the spectra of the formulations were compared with those of the free molecules, changes in relative intensity and slight shifts in the amide and carbonyl bands were noted, suggesting intermolecular interactions between R-PE, 5-FU, and the lipid matrix. These spectral shifts are indicative of hydrogen bonding and rearrangement of the molecular microenvironment, phenomena commonly associated with the encapsulation process, as previously reported for protein–lipid systems [67–68].

Similar alterations have been described in liposomal formulations containing therapeutic agents and biomolecules, where spectral changes were consistent with stabilization of the encapsulated compounds and reduced molecular mobility within the lipid bilayer [67]. Specifically for 5-FU, Ezekiel et al. (2021) [66] confirmed the preservation of its characteristic bands after encapsulation in soybean lecithin liposomes, reinforcing that the presence of these peaks in our formulations indicates successful incorporation. Likewise, Udofot et al. (2015) [64] reported subtle FTIR spectral changes in pH-sensitive liposomes loaded with 5-FU, attributing these modifications to drug–lipid interactions that enhance the stability of the delivery system.

Taken together, these results are consistent with previous literature and demonstrate that both R-PE and 5-FU were effectively incorporated into the nanocarriers. Moreover, the observed spectral modifications suggest that the lipid bilayer not only acted as a physical barrier to immediate diffusion but also provided a stabilizing microenvironment that preserved the structural and functional integrity of the bioactive compounds.

### 3.5 Stability of nanoparticles in serum

Assessing colloidal stability in bovine serum is a critical parameter for inferring the in vivo behavior of nanostructured systems, as the presence of plasma proteins can induce adsorption on the particle surface, aggregation, or changes in surface charge [28]. In this study, liposomes and immunoliposomes containing or not 5-FU and R-PE were incubated in 10% bovine serum for up to 48 h, and the parameters of size, polydispersity index, and zeta potential were monitored.

In general, conventional liposomes exhibited greater colloidal stability, with slight variations in hydrodynamic size and PDI throughout the incubation period. This behavior suggests that the lipid composition employed provided an efficient steric barrier, minimizing nonspecific interactions with serum proteins. The presence of the PEG polymer is known to reduce protein corona formation and confer greater stability in biological media [69–70].

In immunoliposomes, a tendency for size and PDI to increase after 24–48 h of incubation was observed, suggesting possible adsorption of serum proteins and/or reorganization of antibody chains on the surface. This effect is consistent with the literature describing immunoliposomes as more susceptible to interactions with plasma proteins due to the presence of exposed protein domains [71].

The incorporation of 5-FU and R-PE did not promote instability, although small changes in zeta potential were recorded. The shift in zeta potential to less negative values after incubation indicates partial neutralization of surface charges by the serum components, a typical phenomenon in physiological environments. This change, however, did not significantly compromise the physicochemical stability of the formulations, as PDI values remained within the acceptable range (<0.3 for monodisperse systems) [72].

Therefore, the results demonstrate that the developed formulation presents adequate stability under conditions that simulate the biological environment, an essential requirement for in vivo applications. The presence of PEG in the lipid composition and the control of the initial size are probably important factors in maintaining stability, even after 48 h of exposure to bovine serum.

**Figure 6:**
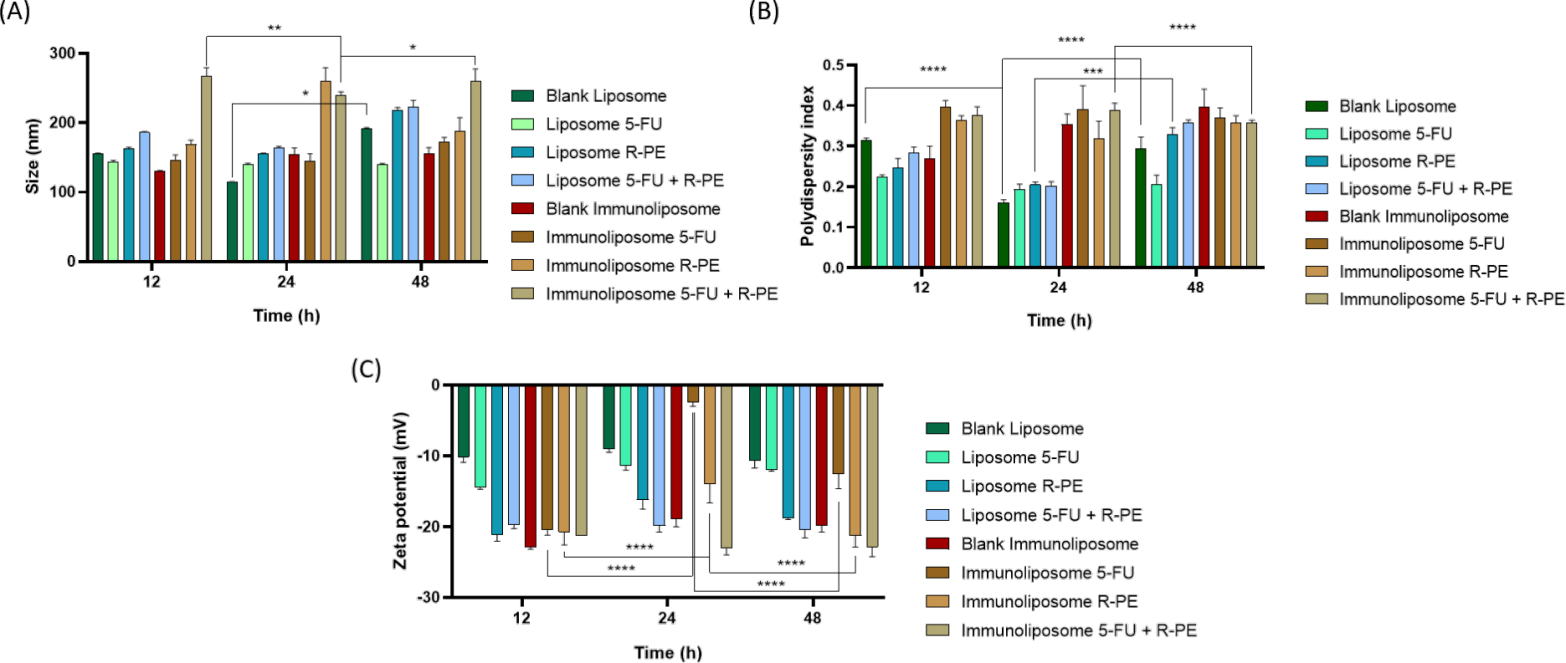
Physicochemical parameters of liposome and immunoliposome formulations during stability in serum study at 4 °C for 48 h. (A) Particle size, (B) PDI, and (C) zeta potential (Two-way-ANOVA with Tukey’s test ****p < 0.001).

### 3.6 In vitro release

The release profiles of 5-FU and R-PE were evaluated using the Korsmeyer–Peppas model (Table 5).

**Table 5:** Kinetic parameters obtained from the application of the Korsmeyer–Peppas model to the release profiles of 5-FU and R-PE. The table presents the determination coefficient (*R*^2^), root mean square error (RMSE), release rate constant (*k*), and release exponent (*n*) values corresponding to the solution, liposome, and immunoliposome formulations.

	5-FU			R-PE		

	Solution	Liposome	Immunoliposome	Solution	Liposome	Immunoliposome

*R* ^2^	0.994	0.984	0.926	0.986	0.997	0.991
RMSE	0.281	0.459	0.839	6.134	1.714	4.661
*k*	9.238	9.669	7.880	117.072	93.085	106.271
*n*	0.040	0	0	0.072	0.012	0.082

For 5-FU, the *R*^2^ values ranged from 0.926 to 0.994, with RMSE between 0.281 and 0.839, indicating excellent correlation between the experimental and calculated data. The kinetic constants (*k*) ranged from 7.880 to 9.669, while the release exponents (*n*) remained low (0.00 to 0.04).

Similarly, for R-PE, high *R*^2^ values (0.986–0.997) and low RMSE values (1.714–6.134) were observed, with *k* ranging from 93.085 to 117.072 and *n* between 0.012 and 0.082.

According to Kozik et al. (2023), *n* ≤ 0.43 values are characteristic of a Fickian release mechanism, in which diffusion is the main process responsible for the release of the active compound through the liposomal matrix. Thus, the obtained low *n* values indicate that both 5-FU and R-PE follow a predominantly diffusional release process, consistent with the behavior described for liposomal systems [23].

In addition, as reported by de Jesús Martín-Camacho et al. (2023), the parameter *k* represents the release constant and reflects the rate at which the compound is released from the system. Higher *k* values correspond to a faster release, whereas lower values indicate a more controlled profile. Therefore, the formulations containing R-PE, which showed higher *k* values (93–117), exhibited a more pronounced release rate compared with the 5-FU formulations (*k* ≈ 7–9), possibly due to the hydrophilic nature of R-PE and its lower interaction with the lipid matrix [23,73].

These results are consistent with the literature, demonstrating that the Korsmeyer–Peppas model adequately describes the release kinetics of the formulations, revealing a sustained and controlled release profile, mainly influenced by diffusion through the liposomal matrix [23,73].

#### 3.6.1 In vitro release of 5-FU

The 5-FU solution showed substantially higher release percentages compared to the nanoformulations at all time points (0.5–24 h; *p* < 0.0001) as shown in Figure 2D. Between 0.5 h and the subsequent times (2–24 h), a significant increase was observed (*p* < 0.0001), whereas no differences were detected between 2, 4, 6, 8, and 24 h (ns), indicating a plateau after 2 h.

The release profile observed for the 5-FU solution evidenced rapid drug diffusion, reaching approximately 70% at 0.5 h and remaining stable around 80% up to 24 h. This behavior was expected, given the hydrophilic character and low molecular weight of 5-FU, which favors its immediate dissolution in an aqueous medium. Similar results were reported by Wang et al. (2022) [74], who highlighted the high release rate of free 5-FU compared to controlled-release systems.

Both 5-FU liposomes and immunoliposomes exhibited controlled and low release (≈15–22%), significantly lower than the solution at all times (*p* < 0.0001). Within each nanoformulation, liposomes remained stable over time (ns). In immunoliposomes, modest increases were observed from 2 to 8 h (*p* < 0.05) and from 2 to 24 h (*p* < 0.05); other comparisons were not significant. Both liposome and immunoliposome formulations demonstrated a significantly lower and sustained release (≈15–22%) throughout the 24 h period.

Direct comparison between liposomes and immunoliposomes was mostly not statistically different (0.5, 8, and 24 h). However, immunoliposomes showed slightly higher release at 2 h (*p* < 0.001), 4 h (*p* < 0.01), and 6 h (*p* < 0.001). In summary, the solution promoted rapid and high release, while the nanoformulations provided sustained and low release, with punctual differences in favor of immunoliposomes between 2–6 h and temporal stability in liposomes. This controlled profile is consistent with other studies that identified liposomal encapsulation as an efficient strategy to prolong 5-FU availability, reduce plasma peaks, and minimize adverse effects [75–76]. Li et al. (2024) [77] also demonstrated that lipid composition, particularly the presence of cholesterol, plays a critical role in decreasing bilayer permeability, thereby slowing drug diffusion [78].

Statistical analysis confirmed significant differences between the 5-FU solution and both liposomal formulations at all evaluated time points (*p* < 0.0001). These findings reinforce that the incorporation of 5-FU into lipid vesicles creates a physical barrier to immediate release, which can be clinically exploited for sustained-release regimens. In contrast, no statistically relevant differences were found between liposomes and immunoliposomes at most time points, suggesting that antibody conjugation to the surface did not significantly alter the in vitro release pattern. This result was also reported by Petrilli et al. (2017) [21], who observed similar release profiles between 5-FU liposomes and immunoliposomes, with more notable differences only in studies of cellular internalization and antitumor efficacy [79].

Although the in vitro release was not modified by the presence of antibodies, immunoliposomes have the recognized advantage of active targeting and enhanced cellular uptake. Previous studies with cetuximab-conjugated immunoliposomes, for example, showed a significant improvement in intracellular delivery of 5-FU and a consequent stronger cytotoxic effect in EGFR-positive tumor cells [21]. Similarly, Scavo et al. (2020) [79] demonstrated that immunoliposomes decorated with antibodies enhanced tumor delivery of 5-FU in sarcoma models, even without differences in in vitro release kinetics.

#### 3.6.2 In vitro release of R-PE

The R-PE release profiles markedly differed among the three tested formulations (Figure 2E). The R-PE solution exhibited significantly higher release at all time points (1–72 h; *p* < 0.0001). A progressive increase was observed between 1 and 24 h (*p* < 0.0001), followed by a plateau from 24 to 72 h (ns). This behavior was expected because R-PE is a hydrophilic protein that rapidly diffuses in aqueous media without structural barriers. Similar findings have been reported for free phycocyanin, which undergoes fast degradation and diffusion compared to encapsulated systems [80].

In contrast, R-PE liposomes displayed a slower and more sustained release, reaching a maximum of ≈80% at 72 h. Significant release was observed only up to 6 h (*p* < 0.0001), after which the profile stabilized and reached a plateau until 72 h. This stable behavior suggests that the lipid bilayer acts as a diffusion barrier for the protein, a phenomenon also reported in liposomal systems containing phycocyanin, where cholesterol content and membrane organization reduced permeability and prolonged pigment availability [81–82].

The R-PE immunoliposome showed an intermediate profile, with release significantly higher than that of liposomes at all time points (*p* < 0.0001), but still lower than that of the solution (*p* < 0.0001). A gradual and continuous increase was observed from 1 to 48 h (*p* < 0.0001), followed by a plateau between 48 and 72 h (ns). These results indicate that antibody conjugation to the vesicle surface may have partially altered the bilayer organization, allowing for slightly greater diffusion. Similar patterns have been described for functionalized immunoliposomes, where surface biomolecules influence stability and release kinetics [83].

The sustained and gradual release observed for both liposomes and immunoliposomes is consistent with previous findings on nanoencapsulated microalgae proteins. Pereira Martins et al. (2023) [15] demonstrated that encapsulation of R-PE in polymeric nanoparticles drastically reduced its release rate compared to that of the free protein, extending its stability in aqueous medium. Similarly, Frota Reis et al. (2025) [24] showed that polymer composition plays a critical role in modulating R-PE release kinetics, with less permeable formulations producing more sustained profiles. Although the systems described in these studies are polymeric, while the present work focuses on liposomal formulations, the results converge in highlighting nanoencapsulation as an effective physical barrier against immediate protein diffusion.

Furthermore, Kopp et al. (2017) [84] demonstrated that the mere presence of free R-PE does not ensure biological efficacy, as the protein undergoes rapid intracellular degradation following internalization. Thus, although the present results indicate that antibody conjugation does not markedly alter in vitro release, immunoliposomes may represent a future clinical advantage by combining a sustained-release profile with active targeting and enhanced cellular uptake. Recent reviews have also emphasized that R-PE bioconjugates exhibit superior stability and more robust functional properties compared to that of the free protein [85].

The results show that the free solution promotes rapid and high release, liposomes provide sustained and stable release, and immunoliposomes maintain this sustained profile but with a slightly higher release than that of liposomes. These findings, in agreement with the literature, reinforce the importance of nanoencapsulation strategies to enhance the stability and modulate the release kinetics of R-PE, while also highlighting the potential of immunoliposomes for therapeutic and bioimaging applications.

### 3.7 Phototoxicity and cytotoxicity

**Figure 7:**
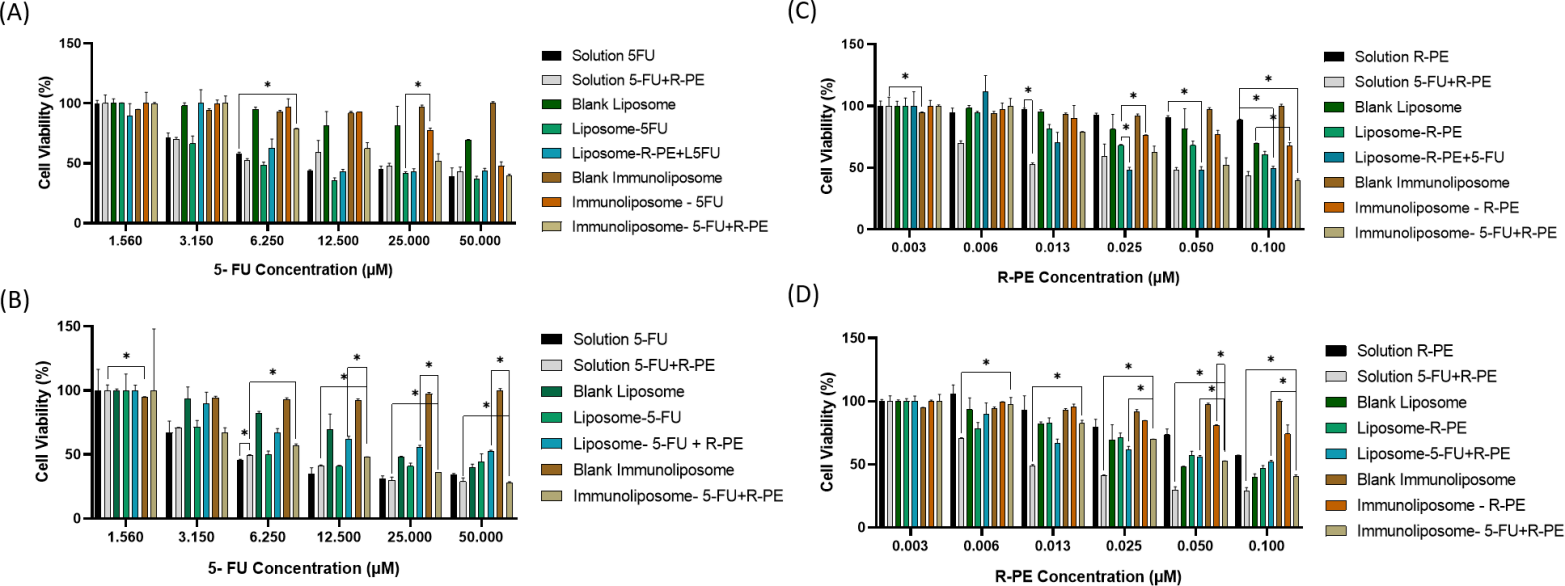
Cellular viability of the formulations in the HCT-116 cell line. (A) Cytotoxicity at different 5-FU concentrations. (B) Phototoxicity at different 5-FU concentrations. (C) Cytotoxicity at different R-PE concentrations. (D) Phototoxicity at different R-PE concentrations.

The cytotoxic effect of the selected liposomal formulation with the lipid HSPC 50, with R-PE and 5-FU, as well as the HSPC IM-07 immunoliposome with R-PE and 5-FU were tested by evaluating the inhibition of cell growth in the CRC HCT-116 cell line. Figure 7 and Table 6 show the results in the absence and presence of light irradiation.

**Table 6:** IC_50_ in the cytotoxicity and phototoxicity evaluations of the formulations and the compounds 5-FU and R-PE evaluated in the HCT-116 cell line, with the respective confidence intervals.

Group	Cytotoxicity		Phototoxicity	

	IC_50_ (µM)	Confidence interval (µM)	IC_50_ (µM)	Confidence interval (µM)

Solution 5-FU	6.914	5.537 to 8.609	–	–
Solution R-PE	0.424	0.220 to 1.512	0.055	0.037 to 0.098
Solution 5-FU/R-PE (IC_50_ 5-FU)	6.812	5.592 to 8.263	3.659	2.478 to 4.969
Solution 5-FU/R-PE (IC_50_ R-PE)	0.0131	0.011 to 0.016	0.007	0.005 to 0.010
Liposome 5-FU	5.479	4.483 to 6.646	–	–
Liposome R-PE	0.331	0.181 to 1.086	0.069	0.048 to 0.121
Liposome 5-FU/R-PE (IC_50_ 5-FU)	10.010	8.162 to 12.280	–	–
Liposome 5-FU/R-PE (IC_50_ R-PE)	0.020	0.016 to 0.025	0.017	0.013 to 0.024
Immunoliposome 5-FU	44.44	32.77 to 73.10	–	–
Immunoliposome R-PE	1.307	1.252 to 1.404	1.390	1.336 to 1.480
Immunoliposome 5-FU/R-PE (IC_50_ 5-FU)	27.63	20.10 to 41.78	–	–
Immunoliposome 5-FU/R-PE (IC_50_ R-PE)	0.07842	0.05269 to 0.1463	0.06562	0.05478 to 0.08122

It was found that the toxicity of 5-FU is similar across all groups, being slightly more toxic in the liposome formulation with 5-FU, reaching the IC50 at a concentration 0.79 times lower than that in solution. The opposite occurred in the formulation with liposomes containing 5-FU and R-PE, since IC_50_ was reached at a concentration 1.4 times higher than the solution with the free drug. Therefore, R-PE may be influencing the release of 5-FU into the intracellular environment. It is noteworthy that the lack of difference in cytotoxicity between the groups with 5-FU in studies on HCT-116 cells may be due to the development of resistance in these cells. Overexpression of the anti-apoptotic tumor protein, translationally controlled by this factor, has already been observed in HCT-116 cells during combined therapy based on 5-FU [86].

The immunoliposomal formulation containing only 5-FU showed an IC_50_ value of 44.44 µM for cytotoxicity, the highest among all formulations analyzed. This result contrasts with that observed for the liposome with 5-FU, whose IC_50_ was 5.47 µM, indicating that, without irradiation, the presence of targeting antibodies did not provide an immediate cytotoxic advantage. However, this difference may be associated with the time required for receptor-mediated internalization, as well as with the more stable nature of the immunoliposome structure, characteristics previously associated with systems functionalized with anti-EGFR antibodies in HCT-116 cells. The high expression of EGFR in this cell line, in fact, is one of the main factors that favor the selective internalization of immunoliposomes, although this process may not be immediate, with time being an important interfering factor [87].

The IC_50_ value obtained for the liposome containing 5-FU (5.5 µM) is within the range observed by other authors for modified liposomal formulations in HCT-116 cells [88]. In contrast, the immunoliposome presented an IC_50_ of 44 µM in the absence of light. This result is not necessarily indicative of worse intrinsic performance, as it may reflect slower internalization kinetics (receptor-mediated) or greater stability/delay in intracellular drug release, as reported by Moreira et al. (2023) [27] and de Sousa et al. (2024) [89]. Previous studies show that immunoliposomes may exhibit less immediate cytotoxicity in vitro if endosome/lysosome-dependent release is slow. In vivo, however, this greater stability may translate into better tumor delivery and lower systemic toxicity [90].

For R-PE, the cytotoxic effect was observed in the presence of 5-FU and was also slightly greater when the molecules were in solution. In the groups with only R-PE, IC_50_ values were not reached for the concentrations tested herein. This corroborates the findings of Sousa et al. (2024), as the IC_50_ for R-PE was found at a concentration of 500 µg/mL [89].

For phototoxicity experiments, a reduction in IC_50_ was found for all groups tested. The R-PE solution revealed a 7.7-fold reduction in IC_50_ upon light irradiation, whereas for R-PE liposomes the reduction was near 4.8 fold. When R-PE was co-administered with 5-FU, the solution provided a higher reduction in IC_50_ compared to that of the liposomes. Previously, it was also observed that the concentration of the photosensitizer also interferes with the cytotoxic effect on the cell, as at the highest concentration the liposome and the R-PE solution (0.1 µM) presented the lowest rate of viable cells. This situation was also observed when comparing phthalocyanine treatments in HCT-116 cells with or without PDT. In this study, the lowest dosage of phthalocyanine to reach the IC_50_ was 0.5 µM, indicating, as in cytotoxicity assays, that R-PE needs to be in a higher concentration, as it is a molecule with hydrophilic properties that will require higher concentrations to reach the IC_50_ value [91].

When we observed the immunoliposomes effect on phototoxicity, the 5-FU immunoliposome in association with R-PE showed a marked reduction in IC_50_, reaching 0.065 µM compared to 0.078 µM for cytotoxicity. Light, in this case, acts as an activating factor, intensifying the action of R-PE and potentiating the cytotoxic effect of encapsulated 5-FU.

The most relevant benefit of the developed system lies in the phototoxic condition (PDT + 5-FU), with an IC_50_ of 0.065 µM for the immunoliposome + 5-FU/R-PE, demonstrating remarkable efficacy and synergism between PDT and active drug release. This result aligns with recent literature showing that encapsulating photosensitizers and combining them with chemotherapeutics in functionalized nanoformulations increases phototherapeutic efficacy and may reduce effective doses [92–93].

This pattern corroborates the data of Chiu et al. (2005) [94] and Song et al. (2020) [95], who reported a significant increase in the sensitivity of HCT-116 cells after exposure to compounds associated with photodynamic stimuli [94]. Furthermore, Ghaddar et al. (2024) [96] reinforce that the combination of localized drug release and induced production of reactive oxygen species (ROS) represents one of the main mechanisms for increasing therapeutic efficacy in light-activation-dependent therapies [96]. Yalçın et al. (2020), in turn, highlights that the encapsulation of photosensitizers in functionalized nanosystems increases the specificity of the therapeutic action and reduces damage to healthy tissues, a scenario consistent with the results obtained for the immunoliposome functionalized with R-PE [97].

The comparison between the formulations with R-PE reinforces this behavior. In the cytotoxicity analysis without light irradiation, both the liposome with R-PE and the immunoliposome with R-PE did not reach the IC_50_ value at the concentrations tested, which was expected for a photosensitizer whose activity depends on light activation. However, after irradiation, the liposome with R-PE reached an IC_50_ of 0.069 µM, while the immunoliposome containing R-PE and 5-FU presented an IC_50_ of 0.065 µM, a value almost twice as low. This significant gain can be attributed to the greater internalization promoted by the functionalization of the system and to the synergistic action between R-PE and 5-FU after activation. This behavior is consistent with the findings of Deng et al. (2023) [98], who demonstrated greater tumor accumulation and photodynamic efficacy of nanoparticles containing photosensitizers in HCT-116 [98]. Similar results were reported by Soriano et al. (2013), who observed increased phototoxicity in liposomal formulations functionalized with m-THPC in colorectal cancer cells [99]. Yalçın et al. (2020) [97] also highlighted that functionalized nanosystems promoted greater production of reactive species and lower toxicity in healthy tissues [97].

Encapsulation of drugs in liposomes and immunoliposomes helps reduce free drug exposure to healthy tissues, increases tumor accumulation (EPR effect) and promotes receptor-mediated internalization, thus lowering the required systemic dose. Moreover, the combination of locally activatable PDT (R-PE) and 5-FU allows more precise tumor targeting via localized irradiation, minimizing toxicity to adjacent tissues [100–101]. Therefore, the data obtained indicate that, although the immunoliposomal formulation containing only 5-FU did not outperform the nonfunctionalized liposome in terms of cytotoxicity, the association with R-PE and the use of light resulted in significantly superior performance. This improvement is directly related to the active targeting capacity of the system, the use of an effective photosensitizer, and the spatiotemporal control provided by light activation – central elements in therapeutic strategies based on multifunctional nanosystems.

Upon light activation, R-PE generates ROS, inducing oxidative damage to lipids, proteins, and DNA and triggering cell death primarily through the intrinsic (mitochondrial) apoptosis pathway and/or necrosis, depending on the ROS load. Recent studies indicate that PDT may also activate autophagy or ferroptosis in specific contexts [102]. In parallel, 5-FU acts as an antimetabolite, inhibiting thymidylate synthase and incorporating into RNA/DNA, triggering replication stress and pro-apoptotic signaling [103]. The combination of both drugs with the targeting agent cetuximab likely induces cell death through the synergy of these mechanisms, with immediate ROS-driven damage accelerating apoptosis and intracellularly released 5-FU exacerbating replicative stress [104]. This dual mechanism can explain the markedly lower IC_50_ observed under phototoxic conditions.

### 3.8 Competitive EGFR-binding of anti-EGFR immunoliposome and EGF in HCT-116 cells using flow cytometry

**Figure 8:**
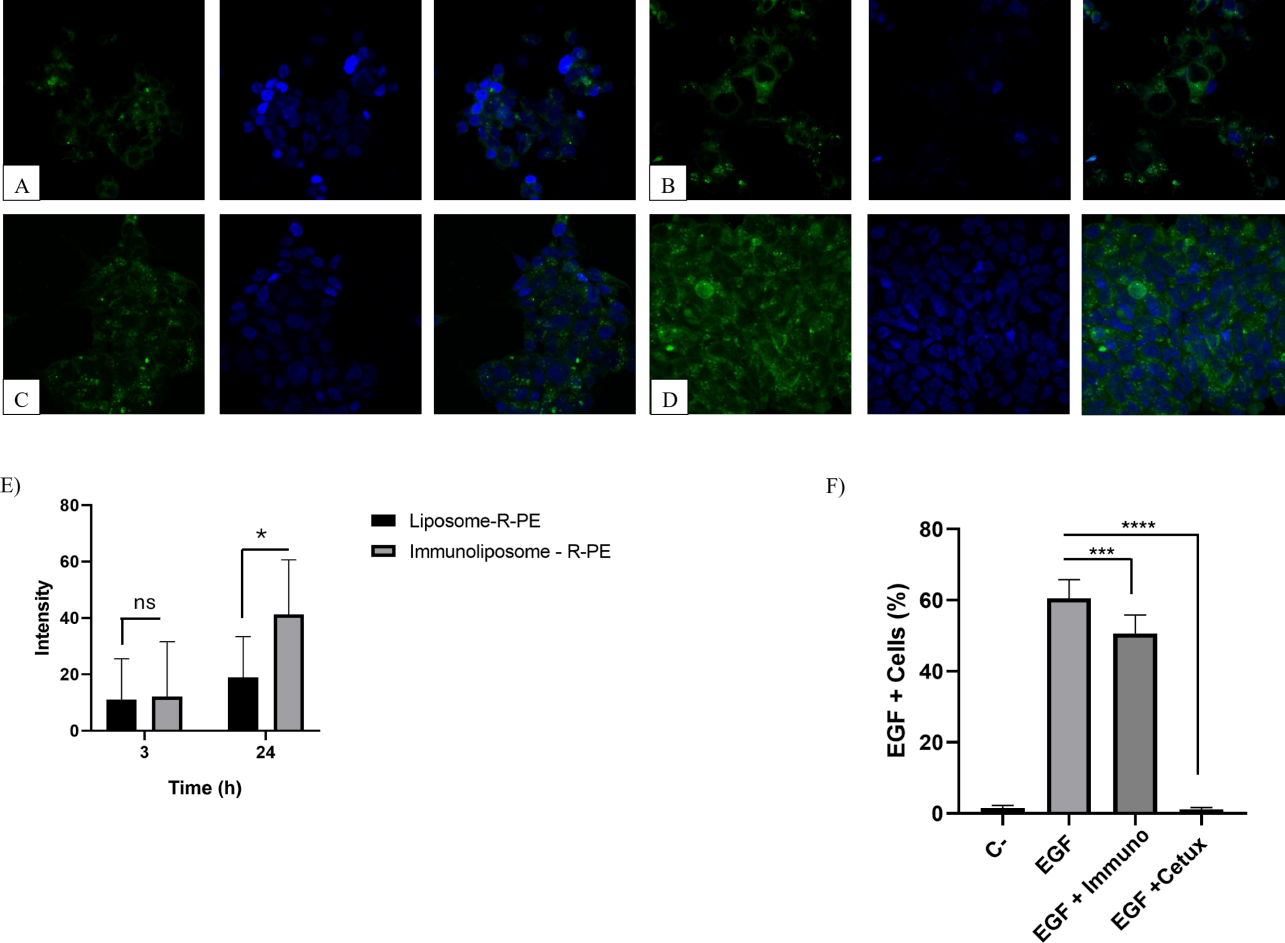
Results of the confocal studies after incubation for 3 h (A and B), and 24 h (C and D) for R-PE liposomes and R-PE immunoliposomes, respectively. The nuclei were stained using DAPI and imaged using a 40× magnification objective. The wavelengths used were 488 nm for excitation and 575–585 nm for emission for R-PE, and 405 nm with emission between 413–472 nm for DAPI. (E) Quantification of R-PE fluorescence intensity after incubation of liposomes and immunoliposomes in HCT-116 cells for 3 and 24 h. (F) Competitive inhibition of EGF binding to EGFR by anti-EGFR immunoliposomes in HCT-116 Cells. C_−_: Negative control, cells treated only with buffer. EGF: Cells treated only with Alexa Fluor™ 488 EGF. EGF+Immuno: Cells treated with HSPC IM 07 and EGF EGF+Cetux: Cells treated with cetuximab and EGF. The values represent the mean ± SD of two independent experiments. Statistical significance was determined using one-way ANOVA followed by Tukey’s multiple comparisons test (ns, not statistically significant; ***p < 0,001, ****p < 0,0001 compared to the EGF treatment group).

The competitive binding of HSPC IM 07 and EGF to EGFR was evaluated in HCT-116 cells using flow cytometry. Treatment with Alexa Fluor™ 488-labeled EGF resulted in approximately 65% of the cell population being positive for EGF binding, indicating robust EGFR expression on the cell surface. In contrast, co-treatment with the anti-EGFR antibody cetuximab led to a marked reduction in EGF-positive cells, with binding levels decreasing to approximately 1% (*p* < 0.0001 vs EGF group; demonstrating effective competition for EGFR binding sites (Figure 8F). Similarly, pre-incubation with anti-EGFR immunoliposomes followed by EGF exposure significantly reduced the proportion of EGF-positive cells from 65% to 55% (*p* < 0.001 vs EGF group), showing that the immunoliposome also competes with EGF for EGFR binding in HCT-116 cells (Figure 8F).

The comparatively lower inhibition by HSPC IM 07 may be attributable to steric hindrance related to the spatial arrangement and density of antibodies or PEG chains on the liposome surface [105–106]. Additionally, variations in internalization pathways including endocytosis, phagocytosis, and macropinocytosis may influence nanoparticle entry into cancer cells, sometimes independently of receptor-mediated pathways [107]. A recent study has shown that anti-EGFR immunoliposomes are internalized via multiple mechanisms, suggesting that direct EGFR competition may not be their exclusive cellular entry route [108]. These findings align with recent literature advocating the potential of immunoliposomes for nanoparticle-mediated drug delivery.

### 3.9 Cellular uptake

The evaluation of the internalization of the R-PE liposome and R-PE immunoliposome formulations, performed by confocal analysis, showed differences over the incubation time. For this, R-PE was used as a fluorescent marker.

In the first time analyzed, 3 h (Figure 8A and B), a similar uptake was observed for both formulations, corroborating the study by Garanina et al. (2024), who reported the same initial peak of internalization, relating this to the endocytosis pathways [109]. According to de Souza et al. 2024, the increase in cellular uptake promoted by cetuximab-functionalized immunoliposomes occurs in a time-dependent manner, being more evident in longer incubation periods. At shorter times, such as up to 3 h, the receptor-mediated internalization process is still ongoing, which may result in confocal microscopy images that do not show significant differences between conventional liposomes and immunoliposomes. Furthermore, the passive internalization of conventional liposomes contributes to this similar initial uptake, masking possible differences in specific EGFR-mediated internalization [52].

At the end of 24 h, the internalization levels increased for both the liposome and the immunoliposome. At this stage, Figure 8C and Figure 8D show an increase for the fluorescence of the immunoliposome, which is higher than that of the liposome, which may indicate that the cellular uptake is dependent on the presence of cetuximab and the incubation time. In the study carried out by Alshaer et al. (2025), better internalization was observed in shorter times, followed by a decrease and subsequent increase, and showing that this is likely due to a common regulation mechanism of the cell, where the cell needs an intermediate time to restart internalization [110]. In studies with EGFR-positive cells (A431 cell line), confocal microscopy revealed higher fluorescence intensities after 24 h of treatment with immunoliposomes, indicating a more efficient cellular uptake of these targeted nanoparticles compared to nonfunctionalized liposomes, corroborating the present study which showed a greater internalization within 24 h in immunoliposomes in EGFR-positive HCT116 cells [19].

Therefore, internalization data showed that the cetuximab-functionalized immunoliposome presented a higher intracellular R-PE signal after 24 h in HCT-116 cells, while at short time points (3 h), the uptake was similar between liposomes and immunoliposomes. This indicates a typical pattern of receptor-mediated internalization: an initial uptake by passive internalization/nonspecific endocytosis, followed by a receptor-dependent increase over time, consistent with several studies describing EGFR-mediated internalization and time-dependent accumulation in EGFR-positive cells [109–110].

The relative increase in fluorescence in immunoliposomes at 24 h corroborates that cetuximab conjugation favors EGFR-dependent recognition and internalization – this is essential for the theranostic strategy since it increases the probability of intracellular delivery of the photosensitizer R-PE and 5-FU to the target site, improving tumor specificity and reducing systemic exposure [111].

Regarding the relevance for the developed system, the observed kinetics suggest that the maximum therapeutic effect (especially the photodynamic effect mediated by R-PE) should be planned considering the time window in which the internalization of immunoliposomes is maximized, a common procedure in PDT studies combined with targeting [101].

The quantitative fluorescence analysis (Figure 8E) corroborated the results qualitatively observed in the confocal images. After 3 h of incubation, no significant differences were found between liposomes and immunoliposomes (*p* > 0.05), indicating similar initial internalization for both formulations. However, after 24 h, a significant increase in fluorescence intensity was observed for R-PE immunoliposomes compared to that of liposomes at the same time point (*p* < 0.05).

These results reinforce that the cellular internalization of immunoliposomes occurs in a time- and antibody-dependent manner, leading to a greater intracellular accumulation of R-PE after 24 h. The alignment between the qualitative and quantitative results demonstrated that cetuximab functionalization enhanced EGFR-mediated recognition and facilitated a more efficient internalization of immunoliposomes than conventional liposomes.

## Conclusion

All the developed liposomes exhibited nanoscale vesicle sizes (above 200 nm) and low polydispersity indices, indicating good homogeneity. The liposomal formulation containing HSPC 50 was selected due to its higher encapsulation efficiency of 5-FU and R-PE, as well as a lower PDI, reflecting greater colloidal stability. These formulations also demonstrated spherical morphology and adequate physicochemical stability. Based on this, immunoliposomes were developed, maintaining nanoscale characteristics and satisfactory polydispersity indices. Among them, the HSPC IM-07 formulation stood out for presenting the highest conjugation efficiency, and was therefore chosen for further studies. In vitro cytotoxicity assays using the HCT-116 colorectal cancer cell line, both the HSPC 50 liposomal formulation and the HSPC IM immunoliposomal formulation containing both molecules showed efficacy comparable to that of free-form drugs. In the presence of light, a significant potentiation of the cytotoxic effect of R-PE was observed, especially when associated with 5-FU in liposomes and immunoliposomes, evidencing a synergistic effect and greater therapeutic efficacy. Thus, the HSPC IM formulation, co-encapsulating R-PE and 5-FU, emerges as a promising alternative. The results obtained through confocal microscopy reinforce the potential of functionalized immunoliposomes for efficient and sustained cellular internalization, suggesting the involvement of a time-dependent regulatory mechanism that favors recycling and intracellular retention of the nanosystems. This behavior is particularly advantageous for applications in phototheranostic therapies, where system selectivity and stability are key determinants of therapeutic success. Moreover, in vitro release assays revealed distinct profiles for 5-FU and R-PE, with liposomes and immunoliposomes promoting sustained and controlled release compared to solutions, thereby reducing premature diffusion and supporting prolonged therapeutic availability. FTIR analyses confirmed the successful incorporation of both molecules into the lipid matrix, as well as intermolecular interactions that likely contributed to their stabilization within the nanosystem. In conclusion, this study highlights the potential of cetuximab-functionalized liposomal systems co-encapsulating R-PE and 5-FU for colorectal cancer therapy, combining selective targeting, photodynamic action, and chemotherapy. These findings advance the field of multifunctional nanocarriers for future clinical applicability of such nanosystems.

## Data Availability

Data generated and analyzed during this study is available from the corresponding author upon reasonable request.
